# Flexible comparative genomics of prokaryotic transcriptional regulatory networks

**DOI:** 10.1186/s12864-020-06838-x

**Published:** 2020-12-16

**Authors:** Sefa Kılıç, Miquel Sánchez-Osuna, Antonio Collado-Padilla, Jordi Barbé, Ivan Erill

**Affiliations:** 1 University of Maryland Baltimore County, Baltimore, MD 21250 USA; 2 Universitat Autònoma de Barcelona, 08193 Bellaterra, Spain

**Keywords:** Comparative genomics, Regulon, Operon, Promoter, Transcription, Bacteria, SOS response, Type III secretion system

## Abstract

**Background:**

Comparative genomics methods enable the reconstruction of bacterial regulatory networks using available experimental data. In spite of their potential for accelerating research into the composition and evolution of bacterial regulons, few comparative genomics suites have been developed for the automated analysis of these regulatory systems. Available solutions typically rely on precomputed databases for operon and ortholog predictions, limiting the scope of analyses to processed complete genomes, and several key issues such as the transfer of experimental information or the integration of regulatory information in a probabilistic setting remain largely unaddressed.

**Results:**

Here we introduce CGB, a flexible platform for comparative genomics of prokaryotic regulons. CGB has few external dependencies and enables fully customized analyses of newly available genome data. The platform automates the merging of experimental information and uses a gene-centered, Bayesian framework to generate and integrate easily interpretable results. We demonstrate its flexibility and power by analyzing the evolution of type III secretion system regulation in pathogenic Proteobacteria and by characterizing the SOS regulon of a new bacterial phylum, the Balneolaeota.

**Conclusions:**

Our results demonstrate the applicability of the CGB pipeline in multiple settings. CGB’s ability to automatically integrate experimental information from multiple sources and use complete and draft genomic data, coupled with its non-reliance on precomputed databases and its easily interpretable display of gene-centered posterior probabilities of regulation provide users with an unprecedented level of flexibility in launching comparative genomics analyses of prokaryotic transcriptional regulatory networks. The analyses of type III secretion and SOS response regulatory networks illustrate instances of convergent and divergent evolution of these regulatory systems, showcasing the power of formal ancestral state reconstruction at inferring the evolutionary history of regulatory networks.

## Background

Transcriptional regulation is the dominant mechanism for regulation of gene expression in bacteria [1, 2]. Transcription factors (TFs) bind promoter regions in sequence-specific manner and can either hinder or promote transcription of target operons containing genes expressed from a shared promoter [2, 3]. Given prior knowledge on the binding specificity of a transcription factor, genomic sequence data can be leveraged to identify putative target sites and reconstruct the transcriptional network, or regulon, under control of a given transcription factor [4, 5]. In theory, this provides the means to elucidate the transcriptional regulatory networks they encode, yielding insights into the molecular mechanisms used by bacteria to orchestrate and coordinate diverse physiological processes. In practice, however, the short and degenerate nature of TF-binding patterns, or motifs, leads to high false positive rates in genome-wide searches, limiting their applicability [6].

Comparative genomics methods for bacterial regulon reconstruction exploit the notion that only functional TF-binding sites should be preserved across substantial evolutionary spans. Hence, the identification of a conserved TF-binding site in the promoter region of two or more orthologous operons should intuitively bolster our confidence in its prediction as a functional element [7–9]. In spite of its applicability and potential, few integrated frameworks have been developed to automate comparative genomics analyses of bacterial regulatory networks using available motif information [10, 11]. Furthermore, several formal and practical aspects of the comparative genomics pipeline remain largely unaddressed. For instance, currently available solutions rely on precompiled databases to predict orthologous operons [12], precluding their use on the vast amount of genomic sequence data representing newly discovered bacterial clades [13–16]. Similarly, formal methods to define what constitutes a functional TF-binding site prediction, and the integration of such predictions across multiple genomes to define what constitutes a conserved binding site, have not been implemented in available tools [17, 18]. Other issues concern the automated integration of multiple sources of experimental information and the generation of interpretable probabilistic results for gene regulation in the light of operon reorganization [19]. Here we present CGB, an integrated pipeline for comparative genomics of bacterial regulatory networks with minimal external dependencies that provides a flexible environment for comparative genomics analyses while introducing a formal probabilistic framework for the integration and interpretation of analysis results. We showcase essential features of CGB through the analysis of HrpB-mediated type III secretion system regulation and in the discovery and validation of a novel TF-binding motif in the Balneolaeota SOS response.

## Results and discussion

### A flexible platform for comparative reconstruction of bacterial regulons

**Fig. 1:**
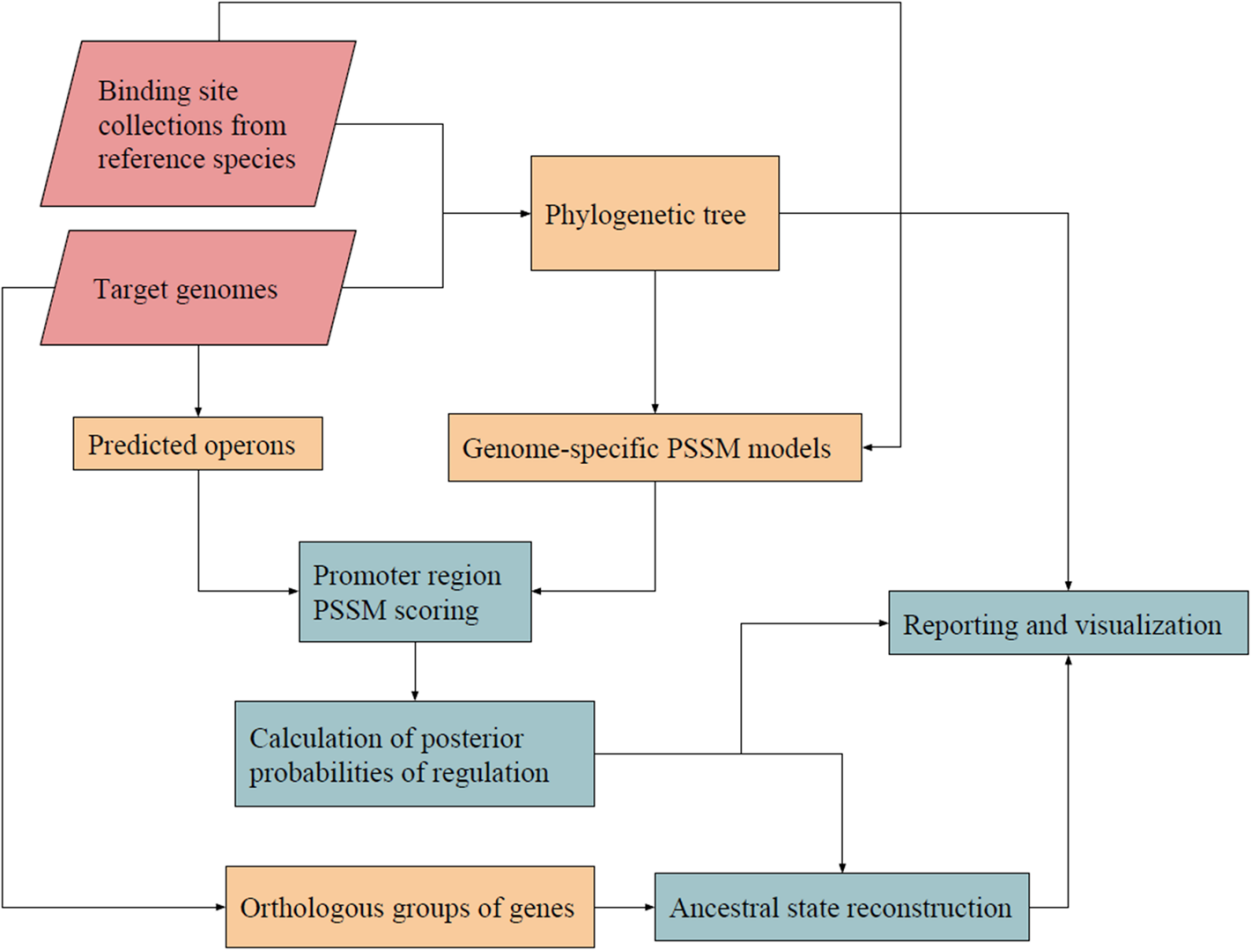
Comparative genomics workflow of CGB. Collections of sites from reference species are combined to build a PSWM model for each target species weighted by its phylogenetic distance to the reference species. Upstream regions of predicted operons are scored and the posterior probability of regulation for each operon is calculated. For each orthologous group of genes, the likelihood of gene regulation in common ancestors are inferred through ancestral state reconstruction

CGB implements a complete computational workflow for the comparative reconstruction of bacterial regulons using available knowledge of TF-binding specificity (Fig. 1). Execution starts with the read-in of a JSON-formatted input file. This file contains the NCBI protein accession number and list of aligned binding sites for at least one transcription factor instance, accession numbers for chromids [20] or contigs mapping to one or more target species, and several configuration parameters. Reference TF-instances are used to detect orthologs in each target genome and a phylogenetic tree of TF instances is generated. The tree is used to combine available TF-binding site information into a position-specific weight matrix (PSWM) for each target species. Operons are predicted in each target species and promoter regions are scanned to identify putative TF-binding sites and estimate their posterior probability of regulation. Groups of orthologous genes are predicted across target species and their aggregate regulation probability is estimated using ancestral state reconstruction methods. CGB outputs multiple CSV files reporting identified sites, ortholog groups, derived PSWMs and posterior probabilities of regulation, as well as plots depicting hierarchical heatmap and tree-based ancestral probabilities of regulation. The following sections describe the novel strategies used to implement the different components of this computational workflow in order to generate an efficient and highly customizable comparative genomics platform.

### Gene-centered, species-specific regulon reconstruction

Previous approaches to regulon reconstruction have focused on the operon as the fundamental unit of regulation [10, 11]. This poses problems for both analysis and reporting due to the frequent reorganization of operons. It is well known, for instance, that after an operon split, genes in the original operon may be regulated by the same transcription factor through independent promoters [2, 3, 21]. CGB uses instead a gene-centered framework, wherein operons become logical units of regulation, but the comparative analysis and reporting of regulons is based on the gene as the fundamental unit of regulation. This enables a rapid assessment of the regulatory state of each gene, while providing the user with detailed information on the operon setup in each organism.

Experimental information on TF-binding specificity is often available in different reference bacterial species, yet the problem of how to transfer and combine this information to target species in a comparative genomics analysis remains largely unaddressed. TF-binding motif information can be transferred across species, but the efficacy of this process decays with evolutionary distance [19]. CGB takes in prior knowledge in the form of a list of TF instances (NCBI protein accessions) in different bacterial strains, together with reported (or inferred) TF-binding sites for each of these TF instances. The collections of TF-binding sites for each TF instance must be aligned, so that the resulting motifs have the same dimensions (i.e. compatible PSWMs). This alignment can be performed manually by the user, or using dedicated tools [22]. CGB automates the transfer of TF-binding motif information from multiple sources across target species. CGB estimates a phylogeny of the reference and target TF orthologs, and uses the inferred distances between reference and target species to generate a weighted mixture PSWM in each target species, following the weighting approach used in CLUSTALW [23]. This provides a principled and reproducible approach for the dissemination of TF-binding motif information, forgoing the need to manually adjust inferred collections of TF-binding sites in each target organism [10].

### Promoter scoring and probabilistic framework

The frequency information in a PSWM can be transformed into a position-specific scoring matrix (PSSM) and used to identify TF-binding sites in genomic sequences. The use of a PSSM score cut-off for predicting putative TF-binding sites in promoter regions has long been the de facto standard in regulon analysis [8–10, 24, 25]. However, this approach is not well-suited for the comparative genomics framework, because thresholds may often need to be tuned in different bacterial genomes owing to their particular distribution of oligomers [6]. To circumvent this problem, here we adopt a Bayesian probabilistic framework originally developed for regulon analysis in metagenomic sequences [26]. This framework estimates posterior probabilities of regulation that are easily interpretable and directly comparable across species.

For each position *i* of a promoter region, we first combine the PSSM scores obtained in the forward (*f*) and reverse (*r*) strands using the function [26]:

1 $$ PSSM\left({s}_i\right)={\log}_2\left({2}^{PSSM\left({s}_i^f\right)}+{2}^{PSSM\left({s}_i^r\right)}\right) $$

To estimate the posterior probability of regulation of a promoter, we define two distributions of PSSM scores within a promoter region. In a promoter not regulated by the TF, we expect a background distribution of scores (*B*). We approximate this distribution using a normal distribution parametrized by the statistics of PSSM scores in promoters genome-wide:

2 $$ B\sim N\left({\mu}_G,{\sigma}_G^2\right) $$

In a promoter regulated by the TF, however, we expect that the distribution of PSSM scores (*R*) be a mixture of both the background distribution (*B*) and the distribution of scores in functional sites. The latter can be approximated with a normal distribution parametrized by the statistics of the TF-binding motif (*M*):

3 $$ R\sim \alpha N\left({\mu}_M,{\sigma}_M^2\right)+\left(1-\alpha \right)N\left({\mu}_G,{\sigma}_G^2\right) $$

The mixing parameter *α* is a prior that corresponds to the probability of a functional site being present in an average-length regulated promoter and can be easily estimated from experimental data. Bacterial transcription factors regulate most of their target genes by binding to a given number of sites in the promoter region, and the average length of the promoter region in a given organism can be readily approximated as the average intergenic distance between the first genes in opposing directons. For a transcription factor known to typically bind one site per regulated promoter, and an estimated average promoter length of 250 bp, we obtain *α* = 1/250 = 0.004. This results in a mixture distribution for the regulated promoter (*R*) drawing predominantly (99.6% of the time) from the background distribution of scores (*B*).

For any given promoter, we can define the posterior probability of regulation *P(R|D)* given the observed scores (*D*):

4 $$ P\left(R|D\right)=\frac{P\left(D|R\right)P(R)}{P(D)}=\frac{P\left(D|R\right)P(R)}{P\left(D|R\right)P(R)+P\left(D|B\right)P(B)} $$

Assuming independence among the scores at each promoter position, the likelihood functions can be estimated for a given score *s*_*i*_ using the density function of the *R* and *B* distributions defined above:

5 $$ P\left(D|R\right)=\prod \limits_{s_i\in D}L\left({s}_i|\alpha N\left({\mu}_M,{\sigma}_M^2\right)+\left(1-\alpha \right)N\left({\mu}_G,{\sigma}_G^2\right)\right) $$

6 $$ P\left(D|B\right)=\prod \limits_{s_i\in D}L\left({s}_i|N\left({\mu}_G,{\sigma}_G^2\right)\right) $$

The priors *P(R)* and *P(B)* can be inferred from the reference collections. *P(R)* can be approximated as the number of known regulated promoters in a reference genome, divided by the total number of operons, and *P(B)* is trivially 1-*P(R)*. Alternatively, the number of regulated promoters can be estimated from the information content of the species-specific TF-binding motif [6, 27].

### Operon prediction

**Fig. 2:**
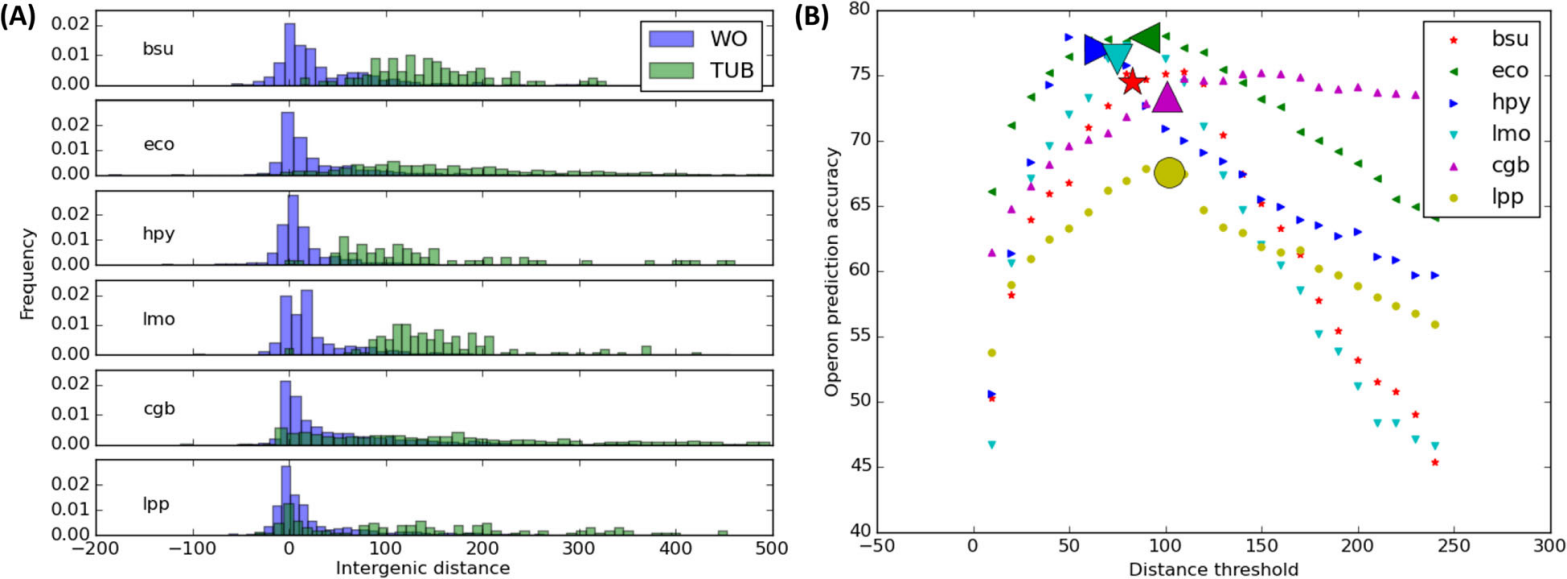
Species-specific intergenic distance threshold for operon prediction. **a** Histograms of intergenic distances of adjacent genes that are on the same strand. WO denotes gene pairs within the same operon. TUB designate gene pairs that define transcription unit boundaries. **b** Percent operon prediction accuracy as a function of intergenic distance threshold (bp). The accuracy of predictions based on the mean intergenic distance is shown using large markers. Accuracy is defined as the ratio of genes that are assigned to their true promoter as annotated in the ODB database. Species acronyms: *Bacillus subtilis* 168 (bsu), *Escherichia coli* MG1655 (eco), *Helicobacter pylori* 26,695 (hpy), *Listeria monocytogenes* EGD-e (lmo), *Corynebacterium glutamicum* ATCC 13032 (cgb) and *Legionella pneumophila* Paris (lpp)

Operon prediction remains a challenging problem in bacterial genomics [28]. Available comparative genomics platforms rely on curated operon databases to improve accuracy, but this limits their applicability to a preselected set of complete bacterial genomes [10, 11]. To enable analyses including newly sequenced, complete or incomplete bacterial genomes, CGB implements a two tiered operon prediction sequence. Intergenic distance is an effective and widely-used predictor of operons. Genes pairs in a same directon (adjacent in the same orientation with no intervening genes in the opposite strand) are considered to belong to an operon if their intergenic distance is below a pre-stablished threshold [29–31]. Because different genomes can have different coding densities, CGB defines this threshold in an adaptive manner as the average intergenic distance in all directons within a given genome. We benchmarked this approach using experimental operon data from the ODB database for six bacterial species (Fig. 2), revealing that dynamically adapting the threshold to each bacterial genome significantly enhances the prediction accuracy.

When reconstructing regulons through comparative genomics, errors in operon prediction can yield two different scenarios: an operon may be split and regulation for some of its genes hence not properly detected, or spurious genes may be incorporated into an operon and their regulation inaccurately predicted. To circumvent this problem CGB predicts operons using a conservative threshold to minimize undesired operon splits (Fig. 2). It then scans the upstream region of all genes to detect any genes within a predicted operon harboring a high-scoring TF-binding site. This is defined as a site scoring above a PSSM score threshold that satisfies the equality between the negative logarithm of the false positive rate (FPR) and the information content of the TF-binding motif [32]. In such cases, the operon is split on the gene with the identified high-scoring site. This allows recovering relevant regulation information for genes that may have inaccurately included within an operon in the initial prediction.

### Ortholog detection and ancestral state reconstruction

Ortholog detection remains a challenging, computationally-intensive problem in bioinformatics [33]. Available comparative genomics platforms make use of precompiled ortholog sets [10, 11], but this restricts the range of bacterial species that can be analyzed. To provide the user with greater flexibility, CGB implements automated detection of orthologous groups in the species under analysis. Orthologous genes are detected as reciprocal best BLAST hits for each pair of species using tBLASTX with default parameters and a 10^− 10^ cutoff e-value. Pairwise reciprocal BLAST hits are used to generate a graph where vertices correspond to gene products and edges denote best reciprocal BLAST hit relationships, and orthologous gene groups are detected as cliques in the graph [34]. Crucially, regulon reconstruction through comparative genomics does not require that all orthologs groups across target species be identified. CGB limits ortholog detection to those genes present in operons with a posterior probability of regulation higher than a user-specified cut-off in any of the target species. This dramatically reduces the complexity of the ortholog detection step, enabling it to be performed in real time.

Integrating the regulon information inferred from each target genome is a critical step in comparative genomics in order to generate insights on the overall makeup of the regulon and its evolutionary history. A common rule of thumb in many comparative genomic analyses has been to assume that the detection of putative TF-binding sites in the promoter region of orthologous operons from two or more sufficiently divergent genomes represents strong evidence of regulation [7–9]. More recently, comparative regulon reconstruction has been formally recast as an ancestral state reconstruction problem, wherein one seeks to infer the likelihood of regulation for a given operon on a phylogenetic tree [18]. CGB implements this approach through bootstrapping ancestral state reconstruction for any given gene on a phylogenetic tree of TF instances. For each bootstrap replicate, CGB assigns discrete regulated (*s*_*1*_) or non-regulated (*s*_*0*_) states to each target species by sampling randomly according to the inferred posterior probability of regulation. If the species does not encode an ortholog for the given gene, the absent (*s*_*a*_) state is assigned. CGB then uses BayesTraits to infer the discrete regulation states on ancestral nodes for each bootstrap replicate [35]. These inferred ancestral states are averaged over all bootstrap replicates to obtain ancestral posterior probabilities of regulation.

### Comparative analysis of the LexA regulatory network in gram-positive bacteria

The SOS response is a transcriptional regulatory network that responds to DNA damage and activates the expression of genes to address DNA lesions and their effects. The SOS response was first described in *Escherichia coli*, where it was shown to regulate over 40 genes involved in three primary functions: DNA repair, inhibition of cell division and translesion synthesis [36, 37]. DNA damage is sensed by the recombination protein RecA, which can promote the autocatalytic cleavage and inactivation of the transcription factor LexA, leading to de-repression of its target genes [36, 38]. Later research has shown that the SOS response is widespread in bacteria but, in contrast with other regulatory networks, multiple LexA-binding motifs have been reported in different bacterial phyla [39]. Both the binding motif and the regulatory network for LexA have been amply documented the Actinobacteria and the Firmicutes [40–44], providing an ideal test case for assessing the performance of CGB. We performed a comparative analysis of the LexA regulon across seven bacterial species: five for which the SOS response has been reported (*Corynebacterium glutamicum* ATCC 13032, *Bacillus subtilis* 168, *Staphylococcus aureus* NCTC 8325, *Listeria monocytogenes* EGD-e and *Mycobacterium tuberculosis* H37Rv) and two related species where the SOS response remains uncharacterized (*Leifsonia xyli* CTCB07, *Acidothermus cellulolyticus* 11B).

**Fig. 3:**
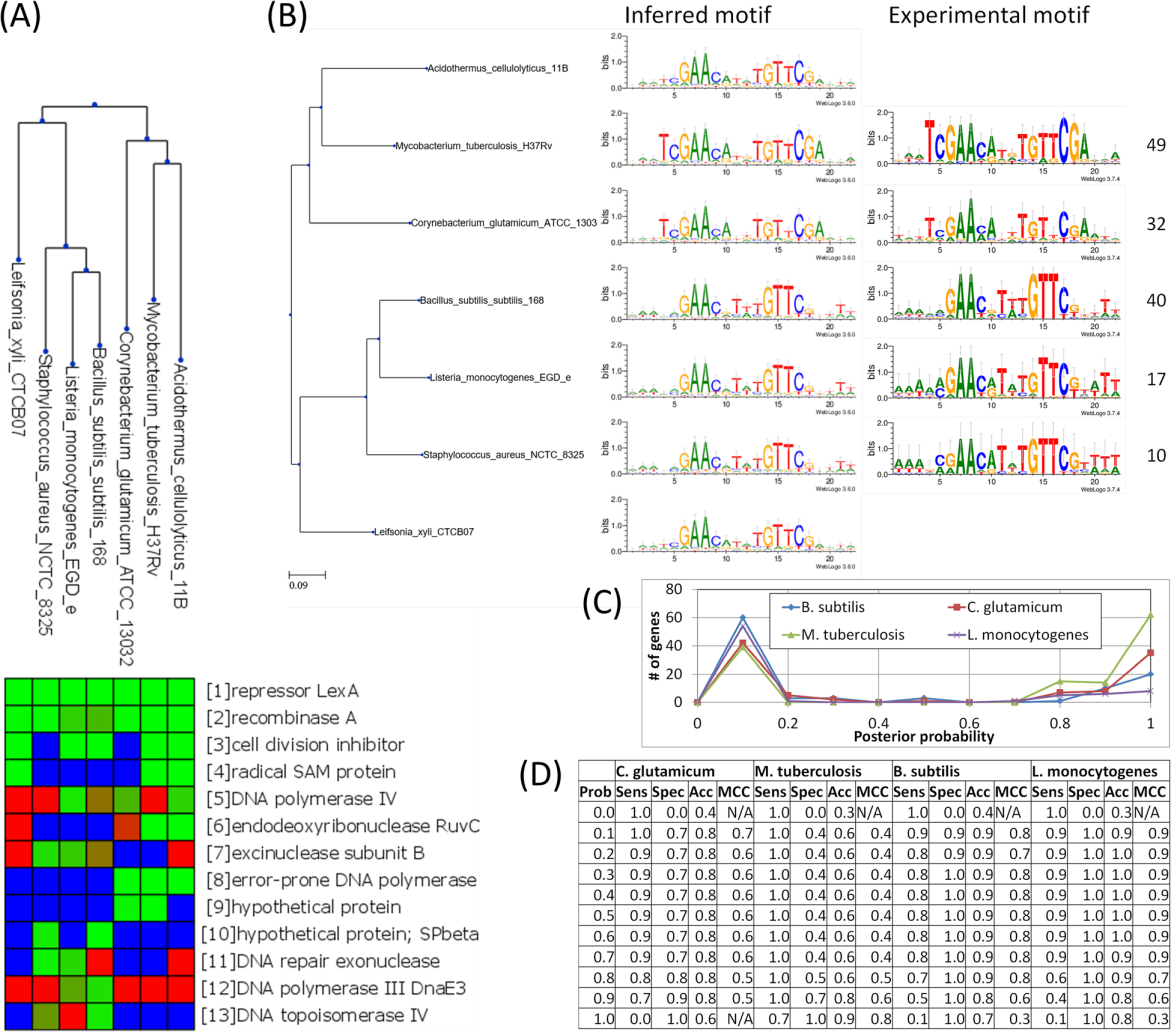
Comparative analysis of the Gram-positive LexA regulon. **a** Partial view of the CGB-generated heatmap depicting the LexA regulon across 7 complete bacterial genomes encompassing members of the Firmicutes and the Actinobacteria. **b** Available experimental motifs (with number of binding sites) and weighted mixture motifs generated by CGB on the target species. **c** Number of genes assigned to different posterior probability of regulation values in different species, among orthologs of genes predicted to be regulated (posterior probability > 0.5) in at least one of the target species. **d** Sensitivity, specificity, accuracy and Matthews Correlation Coefficient (MCC) at different thresholds of posterior probability of binding on different target species

In agreement with previous reports [9], our analysis reveals that the core SOS regulon in Gram-positive bacteria encompasses the LexA and RecA proteins, as well as error-prone polymerases, a radical SAM protein and a cell-division inhibitor (Fig. 3). The plot also illustrates how CGB distributes the available experimental information on TF-binding motifs across all target species, generating phylogenetically weighted mixture motifs that smooth out motifs with low experimental support. We assessed the accuracy of CGB at determining the regulatory state of target genes using the predicted posterior probability of regulation in different reference species. Our results show that, among genes predicted to be regulated in at least one species, the posterior probability generates sharp distinction between regulated and non-regulated genes, yielding accuracy across a broad range of thresholds. Analysis of the prediction accuracy for individual genes reveals that there is a consistent positive correlation (0.33 ± SD 0.08; Spearman correlation coefficient) between true positives in individual species and the number of species in which the gene was predicted to be regulated, supporting the fundamental assumption that conservation of regulatory elements is indicative of functionality.

### Analysis of type III secretion system regulation by HrpB/HrpX

**Fig. 4:**
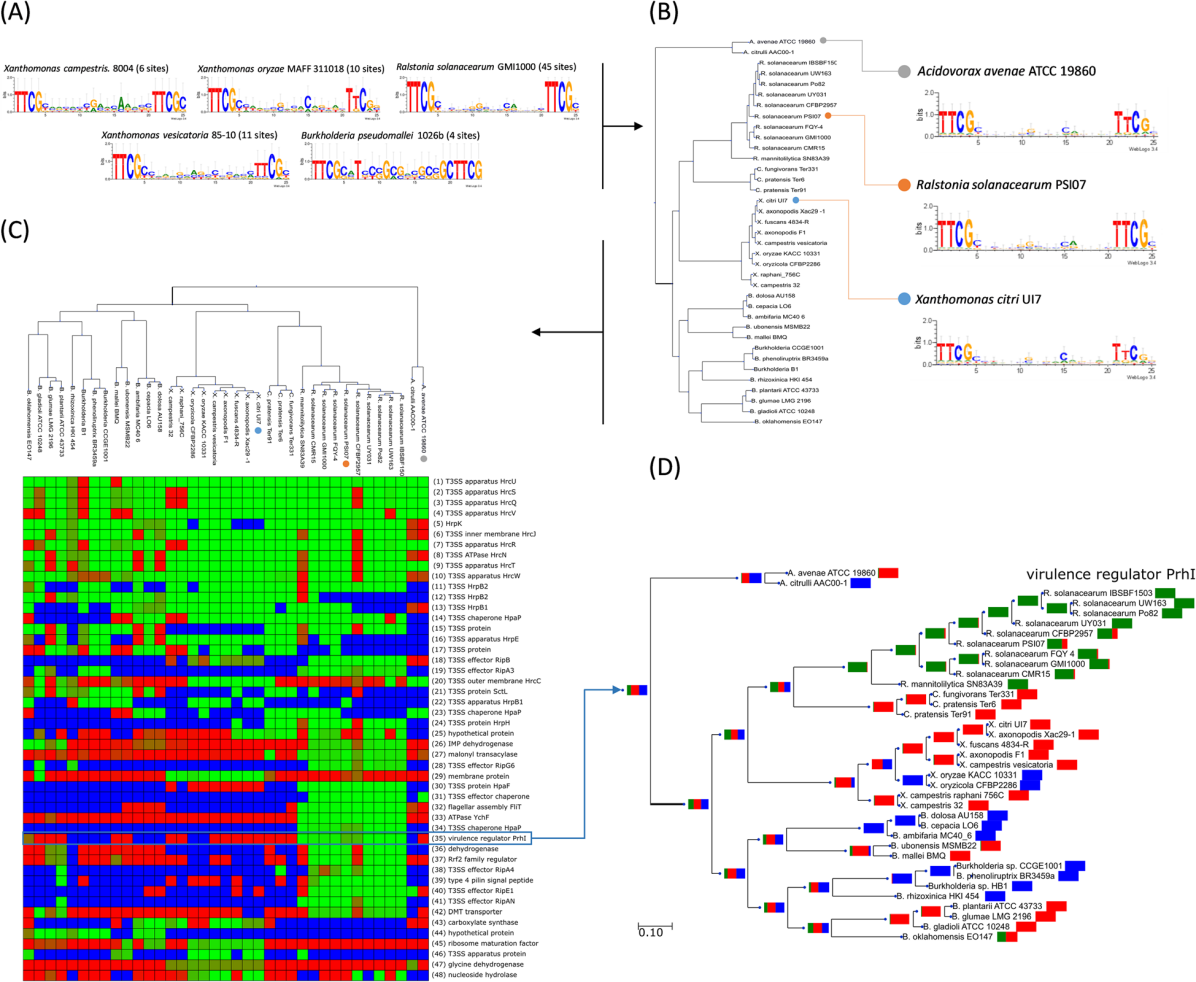
Comparative analysis of the HrpB/X regulon. **a** Experimental HrpB and HrpX motifs used in the analysis, with number of available binding sites indicated. **b** Weighted mixture motifs generated by CGB on different target species following the HrpB/X-based phylogenetic tree. **c** Partial view of the CGB-generated heatmap depicting the HrpB/X regulon across 37 complete bacterial genomes encompassing members of the *Comamonadaceae*, *Burkholderiaceae*, *Oxalobacteraceae* and *Xanthomonadaceae* families. Each row represents an orthologous group, sorted by average posterior probability of regulation. Cells are colored from green (regulation) to red (no regulation); blue denotes ortholog absence. Colored circles next to species names indicate propagated motif (panel B) used in the genome search for the species. **d** Bootstrapped ancestral state reconstruction of the posterior probability of regulation for virulence regulator PhrI

Regulation of the bacterial type III secretion system (T3SS) has been described as mediated by the orthologous transcription factors HrpX (*Xanthomonas oryzae*) and HrpB (*Ralstonia solanacearum*) [45, 46]. In both systems, this TF has been shown to regulate the expression of genes governing the assembly of the T3SS apparatus and several of the effectors that these pathogenic bacteria translocate into host cells. Here we used experimental TF-binding motif from *Xanthomonas* and *Ralstonia* species available in the CollecTF database (Fig. 4a) to analyze the evolution of this regulatory network in several groups of pathogenic bacteria harboring HrpB/X orthologs. This is accomplished by first propagating the experimental motif among all target species (Fig. 4b), and then performing the comparative analysis.

The results (Fig. 4c) reveal that the only elements of the HrpB/X regulon conserved across all the species analyzed are the members of the two core operons that define the structural elements of the type III secretion system. Beyond this core HrpB/X regulon, the analysis (Fig. 4c) shows the divergent uptake of different effectors and chaperones in *Ralstonia*, *Burkholderia* and *Xanthomonas* species, indicating that the core T3SS effectome under HrpB/X regulation in each of these genera diverged soon after each obtained its T3SS module, providing specialized functions in each group [47]. This divergent uptake can be traced to specific lineages through ancestral state reconstruction (Fig. 4d), as in the case of the *Ralstonia* genus for the virulence regulator PrhI [48]. Our results showcase the ability of CGB to automatically disseminate experimental TF-binding motif information among target species in a principled manner (Fig. 4a&b), to use all available sequence files for any given genome (multiple chromosomes and plasmids), to highlight the core elements of a transcriptional regulatory network spanning multiple bacterial orders (Fig. 4c), and to provide a formal inference of the ancestral state of regulation for any given gene (Fig. 4d).

### Reconstruction of the SOS response network in the Balneolaeota phylum

**Fig. 5:**
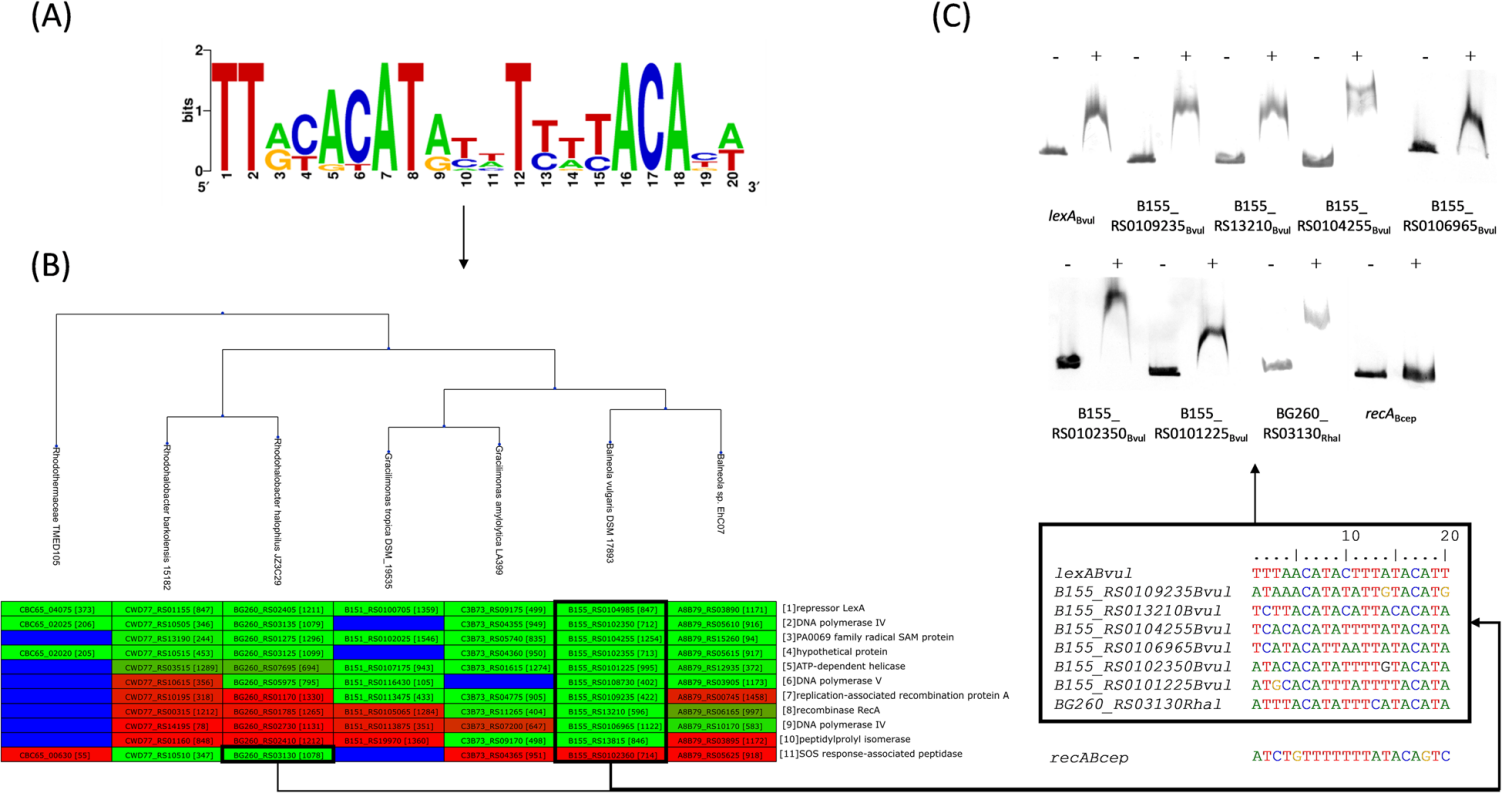
Balneolaeota LexA regulon reconstruction. **a** LexA-binding motif of the Balneolaeota generated by MEME. **b** CGB-generated hierarchical heatmap of posterior probability of regulation for orthologous groups. Each row represents an orthologous group, sorted by average posterior probability of regulation. Cells are colored from green (regulation) to red (no regulation), with blue denoting absence of ortholog. The gene locus tag and operon number are displayed for each ortholog. **c** Sites identified by CGB in the promoter region of genes with orthologs in at least six of the seven species and with average posterior probability greater than 0.5. EMSAs showing binding of purified *Balneola vulgaris* LexA to the promoters fragments of predicted regulated genes containing these sites. The *Burkholderia cepacia recA* promoter is used as negative control. Plus and minus signs indicate presence or absence of LexA in each lane

Metagenomic, single-cell and systematic large-scale genomic sequencing studies have uncovered the existence of several new major bacterial phyla [13–16]. Genomic data corresponding to these phyla is often only available as whole-genome shotgun assemblies and is hence not amenable to comparative genomics studies using available platforms that rely on precompiled complete genome datasets. The Balneolaeota phylum comprises several genera of halotolerant bacteria, but there is scant experimental information on their physiology [49]. Here we coupled motif discovery with comparative genomics using CGB to reconstruct the LexA regulon of the Balneolaeota. Motif discovery (Fig. 5a) was performed on the promoter regions of Balneolaeota LexA homologs with MEME, and the resulting motif was used as input for CGB analysis without phylogenetic weighting. After performing the comparative analysis of the putative LexA regulon across all species with available genome sequence in the Balneolaeota phylum (Fig. 5b), we validated in vitro predicted LexA-binding sites in the promoter region of all genes with orthologs in at least six of the seven species analyzed and presenting an average inter-species posterior probability of regulation above 0.5 (Fig. 5c). The resulting EMSAs on *Balneola vulgaris* and *Rhodohalobacter halophilus* promoters (Fig. 5c) reveal that all the predicted LexA-regulated promoters are bound by LexA. The high precision illustrates the usefulness of leveraging the comparative genomics approach implemented in CGB to boost the accuracy of in silico prediction of TF-regulated genes on individual genomes.

Our results (Fig. 5b) reveal that the Balneolaeota LexA protein binds a novel direct repeat motif with consensus sequence TTACACATATTTTWTACATA (Fig. 5a; Additional File 1). In spite of substantial operon rearrangement, the Balneolaeota LexA regulon encodes a SOS response network encompassing the LexA repressor and the inducer of the system (RecA), as well as several translesion synthesis polymerases (polymerases IV and V), a helicase, a radical SAM protein and a predicted SOS peptidase [50] (Fig. 5c). These results represent the first description of the LexA regulon in the Balneolaeota phylum, reinforcing the notion that translesion synthesis is the primary role of the SOS response and that this system has convergently evolved to regulate error-prone polymerases following drastic changes to the LexA-binding motif [51]. They also illustrate the complex evolutionary history of the LexA protein, which appears to have independently evolved in several instances the ability to recognize a direct repeat motif structure, as opposed to the canonical palindromic LexA-binding motif [51–53]. This example illustrates the ability of CGB to leverage draft genome sequence data to infer regulons in novel phyla, and to operate in tandem with comparative motif discovery methods, using a single instance of the inferred TF-binding motif uniformly distributed across target species.

## Conclusions

Comparative genomics is a powerful method to infer the composition and evolutionary history of prokaryotic transcriptional regulatory networks. The pipeline reported here, CGB, automates the comparative genomics analysis under a formal Bayesian probabilistic framework, enabling the use of ancestral state reconstruction to infer the evolutionary make up of TRNs. The system also enables automated integration of multiple sources of experimental information, forsaking the need to manually port TF-binding motif information. By means of a dynamic operon prediction algorithm and real-time ortholog detection, CGB enables users to analyze any record of draft and complete genomic data available in the NCBI GenBank and RefSeq databases, providing an unparalleled degree of flexibility for this type of comparative analyses. The lack of reliance on external databases, such as operon databases, makes CGB resilient to unexpected downtimes on third-party systems, with the exception of NCBI services. The results reported for two transcriptional regulatory different systems showcase the flexibility of CGB and provide evidence of convergent and divergent evolution in regulatory networks.

## Materials and methods

### CGB platform

CGB is a Python library for comparative genomics of transcriptional regulation in prokaryotes. It is written entirely in Python 2.7 using the object-oriented programming paradigm and deployed as a conda virtual environment (Continuum Analytics). CGB is freely available under a GPL license on GitHub [54]. CGB external requirements are Clustal Omega [55], NCBI BLAST+ [56] and BayesTraits [35].

### Motif data and motif discovery

Collections of experimentally-validated TF-binding sites for HrpB and HrpX were downloaded from the CollecTF database [57]. HrpB orthologs in Proteobacteria and LexA orthologs in the Balneolaeota phylum were detected as best reciprocal BLAST hits using, respectively, the *Ralstonia solanacearum* HrpB protein [WP_011004170.1] and the *Verrucomicrobium spinosum* DSM 4136 LexA protein [WP_009959117] as a queries and a cut-off e-value of 10^− 30^. The upstream regions (− 250, + 50 bp from predicted translational start site) of genes coding for identified LexA orthologs were downloaded from the NCBI GenBank database and input to MEME for motif discovery using the any number of repetitions (ANR) site distribution and motif width limits of 10–22 bp. CGB configuration files for the analyses here reported are provided as supplementary material (Additional File 2).

### Protein purification and electro-mobility shift assays

The *Balneola vulgaris* DSM 17893 *lexA* gene [B155_RS0104985] was synthesized by ATG:biosynthetics GmbH, Germany, subcloned into the pUA1108 vector [58] and overexpressed in *E. coli* BL21-CodonPlus (DE3)-RIL (Stratagene) cells. The resulting LexA His-tagged protein was purified following a previously described protocol [52]. Electro-mobility shift assays (EMSA) were performed using 100 bp-long DNA probes (Additional File 3). Probes were generated using two complementary synthetic oligonucleotides centered on predicted LexA-binding sites and performing PCR with M13 forward and reverse digoxigenin-labeled oligos, as described previously [59]. EMSAs were carried out on a mixture containing 20 ng of each digoxigenin-marked DNA probe and 40 nM of purified LexA protein [60]. Samples were loaded onto 6% non-denaturing Tris-glycine polyacrylamide gels and digoxigenin-labeled DNA-protein complexes were detected using the manufacturer’s protocol (Roche NimbleGen).

## Supplementary information

**Additional file 1.**

**Additional file 2.**

**Additional file 3.**

## Data Availability

Nucleotide sequences analyzed in the current study are available in the NCBI GenBank repository, https://www.ncbi.nlm.nih.gov/nuccore. TF-binding motifs are available in the CollecTF database, http://www.collectf.org/.

## Abbreviations

ANR: Any Number of Repetitions
BLAST: Basic Local Alignment Search Tool
CGB: Comparative Genomics of Bacteria
CSV: Comma Separated Value
DNA: Deoxyribonucleic Acid
EMSA: Electro-Mobility Shift Assay
FPR: False Positive Rate
GPL: GNU General Public License
JSON: JavaScript Object Notation
MEME: Multiple EM for Motif Elicitation
NCBI: National Center for Biotechnology Information
ODB: Operon DataBase
PCR: Polymerase Chain Reaction
PSSM: Position–Specific Scoring Matrix
PSWM: Position–Specific Weight Matrix
SAM: S-adenosyl-L-methionine
SOS: Save Our Souls
T3SS: Type III Secretion System
TF: Transcription factor
TRN: Transcriptional regulatory network

## References

[1] Ptashne M (2005). Regulation of transcription: from lambda to eukaryotes. Trends Biochem Sci.

[2] Ishihama A (2010). Prokaryotic genome regulation: multifactor promoters, multitarget regulators and hierarchic networks. FEMS Microbiol Rev.

[3] Orphanides G, Reinberg D (2002). A unified theory of gene expression. Cell..

[4] Gelfand MS (1995). Prediction of function in DNA sequence analysis. J Comput Biol.

[5] Osada R, Zaslavsky E, Singh M (2004). Comparative analysis of methods for representing and searching for transcription factor binding sites. Bioinformatics.

[6] Erill I, O’Neill MC (2009). A reexamination of information theory-based methods for DNA-binding site identification. BMC Bioinformatics..

[7] Gelfand MS, Novichkov PS, Novichkova ES, Mironov AA (2000). Comparative analysis of regulatory patterns in bacterial genomes. Brief Bioinformatics.

[8] Tan K, Moreno-Hagelsieb G, Collado-Vides J, Stormo GD (2001). A comparative genomics approach to prediction of new members of regulons. Genome Res.

[9] Cornish JP, Matthews F, Thomas JR, Erill I (2012). Inference of self-regulated transcriptional networks by comparative genomics. Evol Bioinformatics Online.

[10] Novichkov PS, Rodionov DA, Stavrovskaya ED, Novichkova ES, Kazakov AE, Gelfand MS (2010). RegPredict: an integrated system for regulon inference in prokaryotes by comparative genomics approach. Nucleic Acids Res.

[11] Liu B, Zhou C, Li G, Zhang H, Zeng E, Liu Q (2016). Bacterial regulon modeling and prediction based on systematic cis regulatory motif analyses. Sci Rep.

[12] Mao F, Dam P, Chou J, Olman V, Xu Y (2009). DOOR: a database for prokaryotic operons. Nucleic Acids Res.

[13] Rinke C, Schwientek P, Sczyrba A, Ivanova NN, Anderson IJ, Cheng J-F (2013). Insights into the phylogeny and coding potential of microbial dark matter. Nature..

[14] Di Rienzi SC, Sharon I, Wrighton KC, Koren O, Hug LA, Thomas BC (2013). The human gut and groundwater harbor non-photosynthetic bacteria belonging to a new candidate phylum sibling to cyanobacteria. Elife..

[15] Herlemann DPR, Geissinger O, Ikeda-Ohtsubo W, Kunin V, Sun H, Lapidus A (2009). Genomic Analysis of “Elusimicrobium minutum,” the First Cultivated Representative of the Phylum “Elusimicrobia” (Formerly Termite Group 1). Appl Environ Microbiol.

[16] Wu D, Hugenholtz P, Mavromatis K, Pukall R, Dalin E, Ivanova NN (2009). A phylogeny-driven genomic encyclopaedia of Bacteria and Archaea. Nature..

[17] Oberto J (2010). FITBAR: a web tool for the robust prediction of prokaryotic regulons. BMC Bioinformatics..

[18] Bykova NA, Favorov AV, Mironov AA (2013). Hidden Markov models for evolution and comparative genomics analysis. PLoS One.

[19] Kılıç S, Erill I (2016). Assessment of transfer methods for comparative genomics of regulatory networks in bacteria. BMC Bioinformatics.

[20] Harrison PW, Lower RPJ, Kim NKD, Young JPW (2010). Introducing the bacterial “chromid”: not a chromosome, not a plasmid. Trends Microbiol.

[21] Erill I, Campoy S, Mazon G, Barbe J (2006). Dispersal and regulation of an adaptive mutagenesis cassette in the bacteria domain. Nucleic Acids Res.

[22] Lee C, Huang C-H (2013). LASAGNA: a novel algorithm for transcription factor binding site alignment. BMC Bioinformatics.

[23] Thompson JD, Higgins DG, Gibson TJ (1994). CLUSTAL W: improving the sensitivity of progressive multiple sequence alignment through sequence weighting, position-specific gap penalties and weight matrix choice. Nucleic Acids Res.

[24] Munch R, Hiller K, Grote A, Scheer M, Klein J, Schobert M (2005). Virtual Footprint and PRODORIC: an integrative framework for regulon prediction in prokaryotes. Bioinformatics.

[25] Yellaboina S, Seshadri J, Kumar MS, Ranjan A (2004). PredictRegulon: a web server for the prediction of the regulatory protein binding sites and operons in prokaryote genomes. Nucleic Acids Res.

[26] Hobbs ET, Pereira T, O’Neill PK, Erill I. A Bayesian inference method for the analysis of transcriptional regulatory networks in metagenomic data. Algorithms Mol Biol. 2016;11. 10.1186/s13015-016-0082-8. 10.1186/s13015-016-0082-8 PMC4938975 27398089

[27] Schneider TD, Stormo GD, Gold L, Ehrenfeucht A (1986). Information content of binding sites on nucleotide sequences. J Mol Biol.

[28] Taboada B, Estrada K, Ciria R, Merino E (2018). Operon-mapper: a web server for precise operon identification in bacterial and archaeal genomes. Bioinformatics..

[29] Westover BP, Buhler JD, Sonnenburg JL, Gordon JI (2005). Operon prediction without a training set. Bioinformatics..

[30] Price MN, Huang KH, Alm EJ, Arkin AP (2005). A novel method for accurate operon predictions in all sequenced prokaryotes. Nucleic Acids Res.

[31] Chuang L-Y, Chang H-W, Tsai J-H, Yang C-H (2012). Features for computational operon prediction in prokaryotes. Brief Funct Genomics.

[32] Hertz GZ, Stormo GD (1999). Identifying DNA and protein patterns with statistically significant alignments of multiple sequences. Bioinformatics..

[33] Nichio BTL, Marchaukoski JN, Raittz RT (2017). New tools in Orthology analysis: a brief review of promising perspectives. Front Genet.

[34] O’Neill PK, Or M, Erill I (2013). scnRCA: A Novel Method to Detect Consistent Patterns of Translational Selection in Mutationally-Biased Genomes. PLoS ONE.

[35] Pagel M, Meade A, Barker D (2004). Bayesian estimation of ancestral character states on phylogenies. Syst Biol.

[36] Walker GC, Neidhart FC, Ingram JL, Low KB, Magasanik B, Schaechter M, Umbarger HE (1987). The SOS response of *Escherichia coli*. Escherichia coli and Salmonella typhimurium: cellular and molecular biology.

[37] Fernandez De Henestrosa AR, Ogi T, Aoyagi S, Chafin D, Hayes JJ, Ohmori H (2000). Identification of additional genes belonging to the LexA regulon in Escherichia coli. Mol Microbiol.

[38] Harmon FG, Rehrauer WM, Kowalczykowski SC (1996). Interaction of Escherichia coli RecA protein with LexA repressor. II. Inhibition of DNA strand exchange by the uncleavable LexA S119A repressor argues that recombination and SOS induction are competitive processes. J Biol Chem.

[39] Erill I, Campoy S, Barbe J (2007). Aeons of distress: an evolutionary perspective on the bacterial SOS response. FEMS Microbiol Rev.

[40] Cirz RT, Jones MB, Gingles NA, Minogue TD, Jarrahi B, Peterson SN (2007). Complete and SOS-mediated response of Staphylococcus aureus to the antibiotic ciprofloxacin. J Bacteriol.

[41] Durbach SI, Andersen SJ, Mizrahi V (1997). SOS induction in mycobacteria: analysis of the DNA-binding activity of a LexA-like repressor and its role in DNA damage induction of the recA gene from Mycobacterium smegmatis. Mol Microbiol.

[42] Au N, Kuester-Schoeck E, Mandava V, Bothwell LE, Canny SP, Chachu K (2005). Genetic composition of the Bacillus subtilis SOS system. J Bacteriol.

[43] van der Veen S, van Schalkwijk S, Molenaar D, de Vos WM, Abee T, Wells-Bennik MHJ (2010). The SOS response of listeria monocytogenes is involved in stress resistance and mutagenesis. Microbiology..

[44] Jochmann N, Kurze A-K, Czaja LF, Brinkrolf K, Brune I, Huser AT (2009). Genetic makeup of the Corynebacterium glutamicum LexA regulon deduced from comparative transcriptomics and in vitro DNA band shift assays. Microbiology..

[45] Valls M, Genin S, Boucher C (2006). Integrated regulation of the type III secretion system and other virulence determinants in Ralstonia solanacearum. PLoS Pathog.

[46] Xue X, Zou L, Ma W, Liu Z, Chen G (2014). Identification of 17 HrpX-regulated proteins including two novel type III effectors, XOC_3956 and XOC_1550, in Xanthomonas oryzae pv. oryzicola. PLoS ONE.

[47] Saier MH (2004). Evolution of bacterial type III protein secretion systems. Trends Microbiol.

[48] Brito B, Aldon D, Barberis P, Boucher C, Genin S (2002). A signal transfer system through three compartments transduces the plant cell contact-dependent signal controlling Ralstonia solanacearum hrp genes. Mol Plant-Microbe Interact.

[49] Hahnke RL, Meier-Kolthoff JP, García-López M, Mukherjee S, Huntemann M, Ivanova NN (2016). Genome-based taxonomic classification of Bacteroidetes. Front Microbiol.

[50] Aravind L, Anand S, Iyer LM (2013). Novel autoproteolytic and DNA-damage sensing components in the bacterial SOS response and oxidized methylcytosine-induced eukaryotic DNA demethylation systems. Biol Direct.

[51] Erill I, Campoy S, Kılıç S, Barbé J. The Verrucomicrobia LexA-binding motif: insights into the evolutionary dynamics of the SOS response. Front Mol Biosci. 2016;3. 10.3389/fmolb.2016.00033. 10.3389/fmolb.2016.00033 PMC4951493 27489856

[52] Sánchez-Osuna M, Barbé J, Erill I (2017). Comparative genomics of the DNA damage-inducible network in the Patescibacteria. Environ Microbiol.

[53] Mazon G, Campoy S, Erill I, Barbe J (2006). Identification of the Acidobacterium capsulatum LexA box reveals a lateral acquisition of the Alphaproteobacteria lexA gene. Microbiology.

[54] Kiliç S, Erill I. CGB: Comparative genomics of transcriptional regulation in Bacteria, https://github.com/erilllab/cgb, last accessed 2019/02/07. English. https://github.com/erilllab/cgb. Accessed 7 Feb 2019.

[55] Sievers F, Wilm A, Dineen D, Gibson TJ, Karplus K, Li W (2011). Fast, scalable generation of high-quality protein multiple sequence alignments using Clustal omega. Mol Syst Biol.

[56] NCBI Resource Coordinators (2017). Database resources of the National Center for biotechnology information. Nucleic Acids Res.

[57] Kiliç S, White ER, Sagitova DM, Cornish JP, Erill I (2014). CollecTF: a database of experimentally validated transcription factor-binding sites in Bacteria. Nucleic Acids Res.

[58] Mayola A, Irazoki O, Martínez IA, Petrov D, Menolascina F, Stocker R (2014). RecA protein plays a role in the chemotactic response and chemoreceptor clustering of Salmonella enterica. PLoS One.

[59] Campoy S, Fontes M, Padmanabhan S, Cortes P, Llagostera M, Barbe J (2003). LexA-independent DNA damage-mediated induction of gene expression in Myxococcus xanthus. Mol Microbiol.

[60] Sanchez-Alberola N, Campoy S, Barbe J, Erill I (2012). Analysis of the SOS response of Vibrio and other bacteria with multiple chromosomes. BMC Genomics.

