# Supplementary material for: Flexible comparative genomics of prokaryotic transcriptional regulatory networks
Source: BMC Genomics. 2020 Dec 16;21(Suppl 5):466. doi: 10.1186/s12864-020-06838-x (PMC7739468; doi:10.1186/s12864-020-06838-x)
Supplement: Supplementary file 1 — Additional file 1. [file 12864_2020_6838_MOESM1_ESM.pdf]

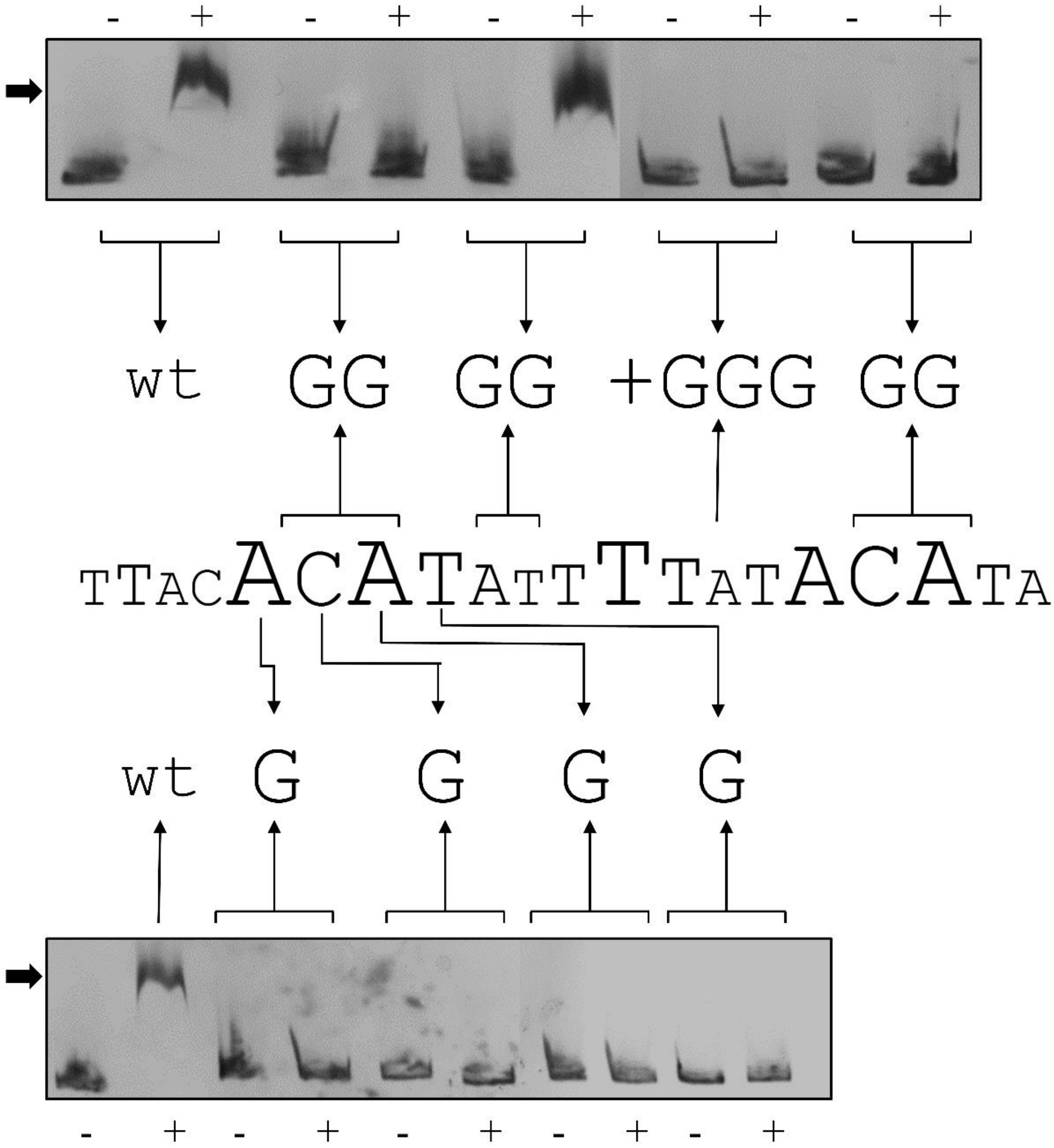

Additional file 1. Site-directed mutagenesis of the *Balneola vulgaris lexA* promoter, introducing mutations that disrupt LexA binding. Arrows from the original sequence to introduced bases indicate nucleotide/pair substitutions. The +GGG stands for the addition of three guanosines, increasing the length spacer region by three base pairs at the designated position.
