# Supplementary material for: Flexible comparative genomics of prokaryotic transcriptional regulatory networks
Source: BMC Genomics. 2020 Dec 16;21(Suppl 5):466. doi: 10.1186/s12864-020-06838-x (PMC7739468; doi:10.1186/s12864-020-06838-x)
Supplement: Supplementary file 2 — Additional file 2. [file 12864_2020_6838_MOESM2_ESM.pdf]

Balneolaeota_LexA_analysis.json


```
{
    "TF": "LexA",
    "motifs": [
        {
           "name": "Gamy_LA399",
            "genome_accessions": ["NZ_PQBS01000035.1"],
            "protein_accession": "WP_103665301.1",
            "sites": ["TTGCACATATCTTTTACACA",
                "TTACACATGTATTTTACATA"]
        },
              {
           "name": "Rhal_JZ3C29",
            "genome_accessions": ["NZ_MDWE01000005.1"],
            "protein_accession": "WP_069130342.1",
            "sites": ["TTGCACATATATCTTACAGT"]
        },
              {
           "name": "Rbar_15182",
            "genome_accessions": ["NZ_PISP01000001.1"],
            "protein_accession": "WP_101071402.1",
            "sites": ["TTACACATATTTCTTACAGT"]
        },
        {
           "name": "Gtro_DSM_19535",
            "genome_accessions": ["NZ_AQXG01000001.1"],
            "protein_accession": "WP_020401662.1",
            "sites": ["TTACACATACCTTTTACACA",
                "TTACACATGTTTCTTACATT"]
        },
        {
           "name": "Bvul_DSM_17893",
            "genome_accessions": ["NZ_AQXH01000001.1"],
            "protein_accession": "WP_018127146.1",
            "sites": ["TTGCACATACTTTATACATT",
                "TTATACATATTTTGTACACA"]
        },
        {
           "name": "B_sp_EhC07",
            "genome_accessions": ["NZ_LXYG01000008.1"],
            "protein_accession": "WP_066218188.1",
            "sites": ["TTGCACATACTTTACACATT",
                "TTATACATGAATCACACACA"]
        },
        {
           "name": "Rbac_TMED105",
            "genome_accessions": ["NHFG01000007.1"],
            "protein_accession": "OUV33799.1",
            "sites": ["TTGTGTATATATTTCACACA"]
        }

    ],
    "genomes": [
      { "name": "Gracilimonas_amylolytica_strain_LA399",
        "accession_numbers": ["NZ_PQBS01000040.1",
          "NZ_PQBS01000039.1",
          "NZ_PQBS01000038.1",
          "NZ_PQBS01000037.1",
          "NZ_PQBS01000036.1",
          "NZ_PQBS01000035.1",
          "NZ_PQBS01000034.1",
          "NZ_PQBS01000033.1",
          "NZ_PQBS01000032.1",
          "NZ_PQBS01000031.1",
          "NZ_PQBS01000030.1",
          "NZ_PQBS01000029.1",
          "NZ_PQBS01000028.1",
```

"NZ_PQBS01000027.1",
"NZ_PQBS01000026.1",
"NZ_PQBS01000025.1",
"NZ_PQBS01000024.1",
"NZ_PQBS01000023.1",
"NZ_PQBS01000022.1",
"NZ_PQBS01000021.1",
"NZ_PQBS01000020.1",
"NZ_PQBS01000019.1",
"NZ_PQBS01000018.1",
"NZ_PQBS01000017.1",
"NZ_PQBS01000016.1",
"NZ_PQBS01000015.1",
"NZ_PQBS01000014.1",
"NZ_PQBS01000013.1",
"NZ_PQBS01000012.1",
"NZ_PQBS01000011.1",
"NZ_PQBS01000010.1",
"NZ_PQBS01000009.1",
"NZ_PQBS01000008.1",
"NZ_PQBS01000007.1",
"NZ_PQBS01000006.1",
"NZ_PQBS01000005.1",
"NZ_PQBS01000004.1",
"NZ_PQBS01000003.1",
"NZ_PQBS01000002.1",
"NZ_PQBS01000001.1"]
},
{
"name": "Rhodohalobacter_halophilus_strain_JZ3C29",
"accession_numbers": ["NZ_MDWE01000092.1",
"NZ_MDWE01000091.1",
"NZ_MDWE01000090.1",
"NZ_MDWE01000089.1",
"NZ_MDWE01000088.1",
"NZ_MDWE01000087.1",
"NZ_MDWE01000086.1",
"NZ_MDWE01000085.1",
"NZ_MDWE01000084.1",
"NZ_MDWE01000083.1",
"NZ_MDWE01000082.1",
"NZ_MDWE01000081.1",
"NZ_MDWE01000080.1",
"NZ_MDWE01000079.1",
"NZ_MDWE01000078.1",
"NZ_MDWE01000077.1",
"NZ_MDWE01000076.1",
"NZ_MDWE01000075.1",
"NZ_MDWE01000074.1",
"NZ_MDWE01000073.1",
"NZ_MDWE01000072.1",
"NZ_MDWE01000071.1",
"NZ_MDWE01000070.1",
"NZ_MDWE01000069.1",
"NZ_MDWE01000068.1",
"NZ_MDWE01000067.1",
"NZ_MDWE01000066.1",
"NZ_MDWE01000065.1",
"NZ_MDWE01000064.1",
"NZ_MDWE01000063.1",
"NZ_MDWE01000062.1",
"NZ_MDWE01000061.1",
"NZ_MDWE01000060.1",
"NZ_MDWE01000059.1",
"NZ_MDWE01000058.1",
"NZ_MDWE01000057.1",
"NZ_MDWE01000056.1",
"NZ_MDWE01000055.1",

"NZ_MDWE01000054.1",
"NZ_MDWE01000053.1",
"NZ_MDWE01000052.1",
"NZ_MDWE01000051.1",
"NZ_MDWE01000050.1",
"NZ_MDWE01000049.1",
"NZ_MDWE01000048.1",
"NZ_MDWE01000047.1",
"NZ_MDWE01000046.1",
"NZ_MDWE01000045.1",
"NZ_MDWE01000044.1",
"NZ_MDWE01000043.1",
"NZ_MDWE01000042.1",
"NZ_MDWE01000041.1",
"NZ_MDWE01000040.1",
"NZ_MDWE01000039.1",
"NZ_MDWE01000038.1",
"NZ_MDWE01000037.1",
"NZ_MDWE01000036.1",
"NZ_MDWE01000035.1",
"NZ_MDWE01000034.1",
"NZ_MDWE01000033.1",
"NZ_MDWE01000032.1",
"NZ_MDWE01000031.1",
"NZ_MDWE01000030.1",
"NZ_MDWE01000029.1",
"NZ_MDWE01000028.1",
"NZ_MDWE01000027.1",
"NZ_MDWE01000026.1",
"NZ_MDWE01000025.1",
"NZ_MDWE01000024.1",
"NZ_MDWE01000023.1",
"NZ_MDWE01000022.1",
"NZ_MDWE01000021.1",
"NZ_MDWE01000020.1",
"NZ_MDWE01000019.1",
"NZ_MDWE01000018.1",
"NZ_MDWE01000017.1",
"NZ_MDWE01000016.1",
"NZ_MDWE01000015.1",
"NZ_MDWE01000014.1",
"NZ_MDWE01000013.1",
"NZ_MDWE01000012.1",
"NZ_MDWE01000011.1",
"NZ_MDWE01000010.1",
"NZ_MDWE01000009.1",
"NZ_MDWE01000008.1",
"NZ_MDWE01000007.1",
"NZ_MDWE01000006.1",
"NZ_MDWE01000005.1",
"NZ_MDWE01000004.1",
"NZ_MDWE01000003.1",
"NZ_MDWE01000002.1",
"NZ_MDWE01000001.1"]
},
{
"name": "Rhodohalobacter_barkolensis_strain_15182",
"accession_numbers": ["NZ_PISP01000007.1",
"NZ_PISP01000006.1",
"NZ_PISP01000005.1",
"NZ_PISP01000004.1",
"NZ_PISP01000003.1",
"NZ_PISP01000002.1",
"NZ_PISP01000001.1"]
},
{
"name": "Gracilimonas_tropica_DSM_19535",
"accession_numbers": ["NZ_AQXG01000048.1",

```
        "NZ_AQXG01000047.1",
        "NZ_AQXG01000046.1",
        "NZ_AQXG01000045.1",
        "NZ_AQXG01000044.1",
        "NZ_AQXG01000043.1",
        "NZ_AQXG01000042.1",
        "NZ_AQXG01000041.1",
        "NZ_AQXG01000040.1",
        "NZ_AQXG01000039.1",
        "NZ_AQXG01000038.1",
        "NZ_AQXG01000037.1",
        "NZ_AQXG01000036.1",
        "NZ_AQXG01000035.1",
        "NZ_AQXG01000034.1",
        "NZ_AQXG01000033.1",
        "NZ_AQXG01000032.1",
        "NZ_AQXG01000031.1",
        "NZ_AQXG01000030.1",
        "NZ_AQXG01000029.1",
        "NZ_AQXG01000028.1",
        "NZ_AQXG01000027.1",
        "NZ_AQXG01000026.1",
        "NZ_AQXG01000025.1",
        "NZ_AQXG01000024.1",
        "NZ_AQXG01000023.1",
        "NZ_AQXG01000022.1",
        "NZ_AQXG01000021.1",
        "NZ_AQXG01000020.1",
        "NZ_AQXG01000019.1",
        "NZ_AQXG01000018.1",
        "NZ_AQXG01000017.1",
        "NZ_AQXG01000016.1",
        "NZ_AQXG01000015.1",
        "NZ_AQXG01000014.1",
        "NZ_AQXG01000013.1",
        "NZ_AQXG01000012.1",
        "NZ_AQXG01000011.1",
        "NZ_AQXG01000010.1",
        "NZ_AQXG01000009.1",
        "NZ_AQXG01000008.1",
        "NZ_AQXG01000007.1",
        "NZ_AQXG01000006.1",
        "NZ_AQXG01000005.1",
        "NZ_AQXG01000004.1",
        "NZ_AQXG01000003.1",
        "NZ_AQXG01000002.1",
        "NZ_AQXG01000001.1"]
    },
  {
        "name": "Balneola_vulgaris_DSM_17893",
        "accession_numbers": ["NZ_AQXH01000018.1",
        "NZ_AQXH01000017.1",
        "NZ_AQXH01000016.1",
        "NZ_AQXH01000015.1",
        "NZ_AQXH01000014.1",
        "NZ_AQXH01000013.1",
        "NZ_AQXH01000012.1",
        "NZ_AQXH01000011.1",
        "NZ_AQXH01000010.1",
        "NZ_AQXH01000009.1",
        "NZ_AQXH01000008.1",
        "NZ_AQXH01000007.1",
        "NZ_AQXH01000006.1",
        "NZ_AQXH01000005.1",
        "NZ_AQXH01000004.1",
        "NZ_AQXH01000003.1",
        "NZ_AQXH01000002.1",
        "NZ_AQXH01000001.1"
```

```
            ]
        },
        {
            "name": "Balneola_sp_EhC07",
            "accession_numbers": ["NZ_LXYG01000020.1",
            "NZ_LXYG01000019.1",
            "NZ_LXYG01000018.1",
            "NZ_LXYG01000017.1",
            "NZ_LXYG01000016.1",
            "NZ_LXYG01000015.1",
            "NZ_LXYG01000014.1",
            "NZ_LXYG01000013.1",
            "NZ_LXYG01000012.1",
            "NZ_LXYG01000011.1",
            "NZ_LXYG01000010.1",
            "NZ_LXYG01000009.1",
            "NZ_LXYG01000008.1",
            "NZ_LXYG01000007.1",
            "NZ_LXYG01000006.1",
            "NZ_LXYG01000005.1",
            "NZ_LXYG01000004.1",
            "NZ_LXYG01000003.1",
            "NZ_LXYG01000002.1",
            "NZ_LXYG01000001.1"]
        },
        {
            "name": "Rhodothermaceae_bacterium_TMED105",
            "accession_numbers": ["NHFG01000001.1",
            "NHFG01000002.1",
            "NHFG01000003.1",
            "NHFG01000004.1",
            "NHFG01000005.1",
            "NHFG01000006.1",
            "NHFG01000007.1",
            "NHFG01000008.1",
            "NHFG01000009.1",
            "NHFG01000010.1",
            "NHFG01000011.1",
            "NHFG01000012.1"]
        }
    ],
    "prior_regulation_probability" : 0.03,
    "alpha" : 0.00285714285714,
    "promoter_up_distance" : 300,
    "promoter_dw_distance" : 50,
    "phylogenetic_weighting": true,
    "site_count_weighting": false,
    "posterior_probability_threshold_for_reporting": 0.5,
    "operon_prediction_probability_threshold": 0.5,
    "operon_prediction_distance_tuning_parameter" : 1.0,
    "ancestral_state_reconstruction" : false,
    "bootstrap_replicates" : 10,
    "heatmap_plot" : true,
    "motif_plot" : true,
    "gene_regulation_plot" : false,
    "taxon_regulation_plot" : false,
    "network_size_plot" : false,
    "site_printout" : true,
    "entrez_email" : "you@email.com"

}
```

Gram_positive_LexA_analysis.json


```
{
    "TF": "LexA",
    "motifs": [
        {
           "name": "LexA_Mtu",
            "genome_accessions": ["NC_000962.3"],
            "protein_accession": "NP_217236.2",
            "sites": [
                "AAATCGAACATGTGTTCGAGTA",
                "GTCTCGAACATGTGTTCGAGAA",
                "GTATCGAACAATTGTTCGATAT",
                "GAATCAAACATGTGTTCGACAG",
                "TATTCGAACATGTATTCGAGTA",
                "GTATCGAATATATTTTCGATGA",
                "GAATCGAACAGGTGTTCGGCTA",
                "AATTCGAACAAGTGATCGAATC",
                "TGTTCGAAAATATGTTCGAAGT",
                "ATCTCGAACATGTGTGCGATAC",
                "ATCTCGAACATACGTTCGAGTA",
                "CATTCGAATATGAGTTCGATCA",
                "TTATCGCACATTCGTTCGATGG",
                "GTATCGAACGATTGTTCGGAAA",
                "GTATAGAACGGTTGTTCGAATA",
                "CATTCGAATATTTGAGCGATGT",
                "TATCCGAACATTTGATCGAAGC",
                "TAGTCACTCATGTGTTCGATAT",
                "TAATCGCTCGCGTGTTCGACAC",
                "CTATCGAACGGGTGTTCTCTCA",
                "TCTTCGAACAGACTTTCGTGCG",
                "GATTCGAACATGTTAGCGAATA",
                "GATTCGAACATGTTAGCGAATA",
                "GTCTCGAACATGTGTTCGAGAA",
                "ATCTCGAACATACGTTCGAGTA",
                "GTATCGAACAATTGTTCGATAT",
                "AAATCGAACATGTGTTCGAGTA",
                "GTCTCGAACATGTGTTCGAGAA",
                "GTATCGAACAATTGTTCGATAT",
                "TACTCGAACGTATGTTCGAGAT",
                "GTATCGAAAGTATGTTCGATCA",
                "GTATCGAAAGTATGTTCGATCA",
                "CCATCGAACGAATGTGCGATAA",
                "TACTCGAATACATGTTCGAATA",
                "ATCTCGAACATGTGTGCGATAC",
                "GTATAGAACGGTTGTTCGAATA",
                "GTATAGAACGGTTGTTCGAATA",
                "GTATCGAACGATTGTTCGGAAA",
                "GAATCAAACATGTGTTCGACAG",
                "GAATCGAACAGGTGTTCGGCTA",
                "TGTTCGAAAATATGTTCGAAGT",
                "GAGTGGAACAGGTGTTCGAAAG",
                "TATTGGAAAATTTGTTCTAAAA",
                "CGTTCGAAAGTGTGAGCGGGTA",
                "CAATCGAATAAATGTTCGAATA",
                "TAATAGAACATATTATCGAACA",
                "ATATTGAAGATATGTACGAGAT",
                "CCTTCGAACATAGCATCGAACA",
                "CCTTCGAACATAGCATCGAACA"
            ]
        },
        {
           "name": "LexA_Cgl",
            "genome_accessions": ["NC_006958.1"],
            "protein_accession": "WP_003857389.1",
            "sites": [
                "TATGCGAACGTTTTTTCTAAAT",
                "TGATCGCAATTGTGTGCTAAAA",
```

```
            "TATTAAAACACTTGTTCTAAAC",
            "TAGTCGAACATGTGAACGGTAT",
            "AATACTGACAGAGGTTCGAATA",
            "ATCTCGAACACTCGTACCATTT",
            "ATTTCGAACAGTTGTGCGTGTA",
            "TATTCGAAAACTTTTCCGATCA",
            "AATTAGAACACTCCTACGAATA",
            "TATTCGAACACGTGAGCGGGTA",
            "TATTCGAACAGTTATTCGATTG",
            "TAGTGGAAAATATGTTCGAGTG",
            "TTTATGAAAATTTGTTTGAGGG",
            "ACTTCGAAAAGTGGCTCCAAGC",
            "CCATCAAACATTTGATCGGTGT",
            "TTATAGAAAACTTGGACGAATC",
            "ACCTCGAACACGTTTGTGTGGA",
            "TTCTCGTAACCTTGTGCGAAAC",
            "AGGGTGGACACTTATTCGAACG",
            "AGTTCGAAAGTATTTCCGAACG",
            "AGTTAGAACATATGTACCACTT",
            "TGATAGAAAATATGTGCTATTT",
            "CTTTTGAATAAGTGTTTGAATA",
            "ATTTTAAAACTGTGTTCGGACA",
            "GAAGCGAACAGAGTTTTTAGTG",
            "TAAGAGAAACGGTGTTCGAAAA",
            "CGATTGAACAAATGTTCGGGGT",
            "TATTCAAATATGTGTTCGAATA",
            "GAGTCGAACAGATGTGTAAATG",
            "GGGGCAAAAATGTGTCCGACCA",
            "GAATAGAAAAAATGTGCGAATA",
            "TCCTCAAAAAAGTGGTCTAATG"
        ]
    },
    {
        "name": "LexA_Lmo",
        "genome_accessions": ["NC_003210.1"],
        "protein_accession": "NP_464827.1",
        "sites": [
            "AAAAAGAATGTATGTTCGCTTT",
            "AAAAAGAATGTATGTTCGCTTT",
            "TGTACGAACGGTTGTTCTATAA",
            "AAAGCGAACATTTATTCGTATT",
            "ATATAGAACATACATTCGATTA",
            "AAAACGAACAAGCGTTCTTATT",
            "GTTGCGAACGTAGGTTCTGTGT",
            "AAAAAGAAAGTGTGTTCGTGTT",
            "TGATAAAACATATGTTCTGTTT",
            "CATACAAACATTTGTTCTTATT",
            "AAACCGAATATACGTTCTTATT",
            "CCACCGAACATATGTTTTTATT",
            "TTCAAGAACGTTTGTTCGTATA",
            "AAAAAGAACGTATGTGCGAAAG",
            "AAACCGAACATATTTTCGCATT",
            "AATAAGAACATTTGTTCGTATA",
            "TTTAAGAACGTTTGTTCGTATA"
        ]
    },
    {
        "name": "LexA_Sau",
        "genome_accessions": ["NC_007795.1"],
        "protein_accession": "WP_001208760.1",
        "sites": [
            "AAATAGAACACGTGTTCGTATA",
            "TTATAGAACATATGTTCGCTTT",
            "AAAGCAAACGTTTGTTCGTAAA",
            "TTTGCGAATATTTGTTCGTATA",
            "AAAACAAACATTTGTTCGTTAA",
            "ATATAGAACATTTGTTCGCTTT",
            "CCTGCAAACGTACGTTCGTTTT",
```

```
            "TATACGAACATGTGTTCTATTT",
            "AAACAGAACATATGTTCGTATT",
            "AACCCGAAAATATGTTTCGTGT"
        ]
    },
    {
        "name": "LexA_Bsu",
        "genome_accessions": ["NC_000964.3"],
        "protein_accession": "NP_389668.1",
        "sites": [
            "TTACAGAACATTTGTTCCTCAC",
            "CATAAGAACTCATGTTCGTGTA",
            "CATAAGAACTCATGTTCGTGTA",
            "ATAAAGAACATTCGTTCTTGTA",
            "TGATAGAACGTATGTTTTGTAT",
            "TATGGGAATGTACGTTCTGGAT",
            "CTTGCGAATGCACGTTCTGTCA",
            "TAAGGGAACGTTTGTTCTATTT",
            "TAAGGGAACGTTTGTTCTATTT",
            "CTTTCGAACGTTTGTTCTGTTT",
            "TTAACGAACGTATGTTTGTAAA",
            "AATCCGAATATGCGTTCGCTTT",
            "AAAAAGAACGTTTGTTCGTATT",
            "AACAGGAATGTTTGTTCGCATT",
            "GACAGAAACGTTTGTTCGTATA",
            "AGTACAAACATAGGTTCGAAAA",
            "TATGAGAACGTATGTTTGTTTA",
            "ACATCGAACTTTTGTTCTGGTT",
            "ATAAGGAACGTTTGTTCTGTTG",
            "AACGAGAACACTTGTTCCCTTT",
            "ATGAAGAACGTTTGTTCTGTTA",
            "AAAAGGAATATTCGTTCGGTAA",
            "TATACGAATTTATGTTCTGTTT",
            "TATGCGAAAGTATGTTCGTTTT",
            "AAAAAGAACTTATGTTCGCTTT",
            "CCTCCAAACTTTTGTTCTTTAT",
            "TAACAGAACGATTGTTCTTATA",
            "AAAGTTAACATATGTTCGCTTC",
            "AACACGAACGTTTGTTTTTCTC",
            "AAAAAGAACATTTGTTTCTTTT",
            "CCACCGAACTTTAGTTCGTATT",
            "AAAAAGAACACTTGTTCTGTTT",
            "ATACCGAACGTATGTTTGCTTT",
            "GTTAGGAATATACGTTCGTATA",
            "CCGGAAAACGTAAGTTCGACTG",
            "TCGTCGAAAACATGTTCGATGA",
            "GTACCGAAACTTTGTTCGGGCA",
            "AAACAGAACATAGGTTCCGCAG",
            "AATAAAAACTTATGTTCGACTT",
            "TTACAGAACATTTGTTCCTCAC"
        ]
    },
    {
        "name": "LexA_Cdi",
        "genome_accessions": ["NC_009089.1", "NC_008226.1"],
        "protein_accession": "WP_003433043.1",
        "sites": [
            "TCTGCAAACATTTGTTCCTGAT",
            "CAAGAGAACAAATGTTTGTAGA",
            "CATAAAAACTTATGTTCTAAAT",
            "GTCAGGAACTAATGTTCGAAAA",
            "AAACAGAACAGTAGTTCCAGAA",
            "GAATAGAACATAAAAATTTTAT",
            "TTCTTGAACTTTAGTTTCTTCT",
            "TTCCTTTTCAGATGTTCTTTTA",
            "CTATAAAATAATAGTTCTGTTA",
            "GTTATGAACTTAGGTTTCTCTC",
            "AGAATGAACTATTGTTTTATTT",
```

```
            "TAGCTGAACCAAAGTTCCAATC",
            "TTTGTGAACCAATGTTTGATAT",
            "AAATGGAACTTTAGTTCTATTT",
            "TTGCAGAACGGGTGTTTTTTGA",
            "TATAGGAACTTTAGTTCTTGGA"
        ]
    }
],

"genomes": [
    {
        "name": "Acidothermus_cellulolyticus_11B",
        "accession_numbers": ["NC_008578.1"]
    },
    {
        "name": "Corynebacterium_glutamicum_ATCC_13032",
        "accession_numbers": ["NC_006958.1"]
    },
    {
        "name": "Bacillus_subtilis_subtilis_168",
        "accession_numbers": ["NC_000964.3"]
    },
    {
        "name": "Staphylococcus_aureus_NCTC_8325",
        "accession_numbers": ["NC_007795.1"]
    },
    {
        "name": "Listeria_monocytogenes_EGD_e",
        "accession_numbers": ["NC_003210.1"]
    },
    {
        "name": "Mycobacterium_tuberculosis_H37Rv",
        "accession_numbers": ["NC_000962.3"]
    },
    {
        "name": "Leifsonia_xyli_CTCB07",
        "accession_numbers": ["NC_006087.1"]
    }
],

"prior_regulation_probability" : 0.03,
"alpha" : 0.00285714285714,
"promoter_up_distance" : 300,
"promoter_dw_distance" : 50,
"phylogenetic_weighting": true,
"site_count_weighting": true,
"posterior_probability_threshold_for_reporting": 0.75,
"operon_prediction_probability_threshold": 0.5,
"operon_prediction_distance_tuning_parameter" : 1.0,
"ancestral_state_reconstruction" : false,
"bootstrap_replicates" : 10,
"heatmap_plot" : true,
"motif_plot" : true,
"gene_regulation_plot" : false,
"taxon_regulation_plot" : false,
"network_size_plot" : false,
"site_printout" : false,
"entrez_email" : "you@email.com",
   "entrez_apikey" : "XXXXXXXXXXXXXX",
   "sleep" : 1.5,
"TF_eval" : 0.00000000001,
"homolog_eval" : 0.1,
"hmmer_eval" : 0.0000001,
"COG_search" : true,
"NOG_search" : false,
"PFAM_search" : false,
"COG_dbname" : "/home/ivan/HMMERdbs/COG_database.hmm",
"eggNOG_dbname" : "/home/ivan/HMMERdbs/bact.hmmer",
```

```
    "PFAM_dbname" : "/home/ivan/HMMERdbs/Pfam-A.hmm",
    "OGejump" : 5,
    "maxCOG" : 2,
    "maxNOG" : 2,
    "maxPFAM" : 2
}
```

HrpBX_analysis.json


```
{"TF": "HrpB",
    "motifs":
       [
        {
                    "name": "HrpX_Xca_8004a",
            "genome_accessions": ["NC_014309.1"],
                    "protein_accession": "WP_013208249.1",
            "sites":
                    [
                            "TTCGCGCGTTTCGCAATTGCCAACC"
                    ]
            },
            {
                    "name": "HrpX_Xca_8004b",
            "genome_accessions": ["NC_003902.1"],
            "protein_accession": "WP_011036364.1",
            "sites":
                    [
                            "TTCGGAGCAATAACCATCCGTTCGC",
                            "TTCGCACATGAAAATAAAGGTTCGC",
                            "TTCGCTAGCTCGCGCAGAGTTTCGC",
                            "TTCGTGTCGTTGCAACGCCCTTCGC",
                            "TTCGTCGTCCCGGTCAATTCTTCGG",
                            "TTCGCCATACCGATGAAGTCTTCGC"
                    ]
            },
            {
                    "name": "HrpX_Xve_85_10",
            "genome_accessions": ["NC_007508.1"],
            "protein_accession": "WP_011346769.1",
            "sites":
                    [
                            "TTCGCGGCGCGCGCGCCAGCTTCGT",
                            "TTCGCACACGCACCCTTGCATTCGC",
                            "TTCGCTTTGCATCGCTGCACTTCGT",
                            "TTCGGCCAAGCTTACGTCAACTCGC",
                            "TTCGTTTTTGGAGCGCCGCGTGCGG",
                            "TTCGTTTGCTGCGGCGGCGCTTCGT",
                            "TTCGCCAGCGAATTCCGATATTCGC",
                            "TTCGCCAGGCCATCCACACATTCGC",
                            "TTCGCCGGACCAGCTATCGCTTCGC",
                            "TTCGCCCATGACCATGCAGCTTCGC",
                            "TTCGCCAAAATAGTTCGTCGGCCAG"
                    ]
            },
            {
                    "name": "HrpX_Xor_MAFF_311018",
            "genome_accessions": ["NC_007705.1"],
            "protein_accession": "WP_011258190.1",
            "sites":
                    [
                            "TTCGCTTAACGCGACCGGTCTGCGG",
                            "TTCGCCAAATCGCACATCGATTCTG",
                            "TTCGCTTGCCCGTTAAGTGTTTCGT",
                            "TTCGTTTACGGAATCGCTTGTTCGT",
                            "TTCGCAAGTTCTGCAGCTTTTTCGG",
                            "TTCGCTTTGCGTCGCTTTACTTCGT",
                            "TTCGCCAGGAGACGCGTAACTGCGG",
                            "TTCGGACTGCGCGACGCCGGTTCGC",
                            "TTCGTCGCCGCACACAGGAATTCAC",
                            "TTCGCCATTGATGACAGAATTTCAC"
                    ]
            },
            {
                    "name": "Hrpb_Rso_GMI1000",
            "genome_accessions": ["NC_003296.1"],
```

```
        "protein_accession": "WP_011004170.1",
        "sites":
                [
                        "TTCGTACGCTTGCACAAGGTTTCGG",
                        "TTCGCGTGCATGACCACGATTTCGG",
                        "TTCGCTTTGAGGTCGGCAACTTCGC",
                        "TTCGTCCCGCGCACGGGACATTCGG",
                        "TTCGGCGGGGGCGCCGGCCCTTCGC",
                        "TTCGCTCGCCGGTCTAGTGCTTCGC",
                        "TTCGGGCCAAATGCGAAACCTTCGC",
                        "TTCGCAATCCCCAACATTGTTTCGC",
                        "TTCGCCGTTCAGGCCGCGGGTTCGC",
                        "TTCGTTTTCCAGCTCGAGTGTTCGG",
                        "TTCGCGAACGGAGACCACTTTTCGC",
                        "TTCGGGCCGGGCCCGATTTCTTCGC",
                        "TTCGCACCGGCGCGCACAACTTCGC",
                        "TTCGTTTTGTGATGTGCCGCTTCGG",
                        "TTCGCATCACCCTTCATTTCTTCGC",
                        "TTCGCAAGGCGGGCGAGGAGTTCGG",
                        "TTCGCACGGGGCCTCCCCAATTCGC",
                        "TTCGCTCGGCACAACAAGACTTCGC",
                        "TTCGCATCTTAACGCATAACTTCGC",
                        "TTCGGTTCGGGCACGACTGCTTCGC",
                        "TTCGCCGGCAGCGCGTCCGGTTCGA",
                        "TTCGCTGCCGGAGGCAGGGCTTCGT",
                        "TTCGCCTGTTGCCGTGTTTTTTCGT",
                        "TTCGTTTGCCGGCAGCTTCTTTCGT",
                        "TTCGTACCTGTCCTCAATGTTTCGC",
                        "TTCGCATACGCGCACATCCTTTCGC",
                        "TTCGTCCAAAGCGTTCGCGGTTCGG",
                        "TTCGCTTTTGGATGCGGCGCTTCGT",
                        "TTCGGTCGCCGCGCGGGCATTTCGC",
                        "TTCGCCCGAACGGCAGCGCGTTCGC",
                        "TTCGGGCGGCGAACCGATCATTCGT",
                        "TTCGTCACCGGGCCGCCACTTTCGC",
                        "TTCGCCCGCGGCCACCGGGCTTCGC",
                        "TTCGTTTATGCGAGCACATTTTCGG",
                        "TTCGCGGATGGACAACGCGCTTCGC",
                        "TTCGCTCGCCGGTCTAGTGCTTCGC",
                        "TTCGCGCGGGCCCGCAGATTTTCGT",
                        "TTCGCCCCCGGCCCCATCCATTCGC",
                        "TTCGCGGCGGGCGACACCGGTTCGT",
                        "TTCGCCAACGCAGCCAGTTTTTCGC",
                        "TTCGTGTTAGCTGTGAAAGCTTCGC",
                        "TTCGCCCCCGGCCCCATCCATTCGC",
                        "TTCGGCTCCGGCCTCATCACTTCGG",
                        "TTCGCTTTTGGATGCGGCGCTTCGT",
                        "TTCGCCCATGGAACCGGCGCTTCGC"
                ]
           },
           {
                "name": "Hrpb_Bps_1026b",
        "genome_accessions": ["NC_006351.1"],
        "protein_accession": "WP_004530623.1",
        "sites":
                [
                        "TTCGGCTGCCGCGACGCCGCTTCGC",
                        "TTCGCATCCGGCGGCGCGGCTTCGG",
                        "TTCGCGTTTCGACTTGCGGCTTCGG",
                        "TTCGCATCCGGCGCGCGCGCTTCGC"
                ]
           }
],
"genomes":
   [
    {
        "name": "R_solanacearum_UY031",
        "accession_numbers": ["NZ_CP012688.1", "NZ_CP012687.1"]
```

```
        },
        {
            "name": "R_solanacearum_PSI07",
            "accession_numbers": ["NC_014310.1", "NC_014311.1", "NC_014308.1"]
        },
        {
            "name": "R_solanacearum_GMI1000",
            "accession_numbers": ["NC_003296.1", "NC_003295.1"]
        },
        {
            "name": "R_solanacearum_IBSBF1503",
            "accession_numbers": ["NZ_CP012944.1", "NZ_CP012943.1"]
        },
        {
            "name": "R_mannitolilytica_SN83A39",
            "accession_numbers": ["NZ_CP011258.1", "NZ_CP011257.1"]
        },
        {
            "name": "C_fungivorans_Ter331",
            "accession_numbers": ["NC_015856.1", "NC_010332.1"]
        },
        {
            "name": "C_pratensis_Ter91",
            "accession_numbers": ["NZ_CP013234.1"]
        },
        {
            "name": "B_cepacia_LO6",
            "accession_numbers": ["NZ_CP011301.1", "NZ_CP011302.1"]
        },
        {
            "name": "B_ambifaria_MC40_6",
            "accession_numbers": ["NC_010557.1", "NC_010553.1", "NC_010552.1", "NC_010551.1"]
        },
        {
            "name": "B_plantarii_ATCC_43733",
            "accession_numbers": ["NZ_CP007212.1","NZ_CP007214.1", "NZ_CP007213.1"]
        },
        {
            "name": "B_gladioli_ATCC_10248",
            "accession_numbers": ["NZ_CP009322.1","NZ_CP009323.1", "NZ_CP009321.1",
"NZ_CP009320.1", "NZ_CP009319.1"]
        },
        {
            "name": "Burkholderia_sp_CCGE1001",
            "accession_numbers": ["NC_015137.1","NZ_CP012899.1", "NC_015136.1"]
        },
        {
            "name": "Burkholderia_sp_HB1",
            "accession_numbers": ["NZ_CP012193.1","NZ_CP012192.1"]
        },
        {
            "name": "B_rhizoxinica_HKI_454",
            "accession_numbers": ["NC_014723.1","NC_014722.1", "NC_014718.1"]
        },
        {
            "name": "B_ubonensis_MSMB22",
            "accession_numbers": ["NZ_CP009488.1","NZ_CP009487.1", "NZ_CP009486.1"]
        },
        {
            "name": "B_oklahomensis_EO147",
            "accession_numbers": ["NZ_CP008726.1","NZ_CP008727.1"]
        },
        {
            "name": "B_mallei_BMQ",
            "accession_numbers": ["NZ_CP008723.1","NZ_CP008722.1"]
        },
        {
            "name": "A_citrulli_AAC00-1",
```

```
            "accession_numbers": ["NC_008752.1"]
        },
        {
            "name": "A_avenae_ATCC_19860",
            "accession_numbers": ["NC_015138.1"]
        },
        {
            "name": "X_citri_UI7",
            "accession_numbers": ["NZ_CP008987.1","NZ_CP008988.1", "NZ_CP008989.1"]
        },
        {
            "name": "X_axonopodis_Xac29_1",
            "accession_numbers": ["NC_020797.1","NC_020798.1", "NC_020800.1", "NC_020801.1"]
        },
        {
            "name": "X_oryzae_KACC_10331",
            "accession_numbers": ["NC_006834.1"]
        },
        {
            "name": "X_campestris_vesicatoria",
            "accession_numbers": ["NC_007504.1","NC_007505.1", "NC_007506.1", "NC_007507.1",
"NC_007508.1"]
        },
        {
            "name": "X_campestris_raphani_756C",
            "accession_numbers": ["NC_017271.1"]
        },
        {
            "name": "B_phenoliruptrix_BR3459a",
            "accession_numbers": ["NC_018695.1", "NC_018696.1", "NC_018672.1"]
        }
    ],
    "prior_regulation_probability" : 0.03,
    "alpha" : 0.00285714285714,
    "promoter_up_distance" : 300,
    "promoter_dw_distance" : 50,
    "phylogenetic_weighting": true,
    "site_count_weighting": true,
    "posterior_probability_threshold_for_reporting": 0.75,
    "operon_prediction_probability_threshold": 0.5,
    "operon_prediction_distance_tuning_parameter" : 1.0,
    "ancestral_state_reconstruction" : true,
    "bootstrap_replicates" : 100,
    "heatmap_plot" : true,
    "motif_plot" : true,
    "gene_regulation_plot" : true,
    "taxon_regulation_plot" : false,
    "network_size_plot" : false,
    "site_printout" : true,
    "entrez_email" : "you@email.com",
       "entrez_apikey" : "XXXXXXXXXXXXXX",
       "sleep" : 0.5,
    "TF_eval" : 0.00000001,
    "homolog_eval" : 0.001,
    "hmmer_eval" : 0.000000001,
    "COG_search" : true,
    "NOG_search" : true,
    "PFAM_search" : true,
    "COG_dbname" : "/home/ivan/HMMERdbs/COG_database.hmm",
    "eggNOG_dbname" : "/home/ivan/HMMERdbs/bact.hmmer",
    "PFAM_dbname" : "/home/ivan/HMMERdbs/Pfam-A.hmm",
    "OGejump" : 5,
    "maxCOG" : 2,
    "maxNOG" : 2,
    "maxPFAM" : 2
}
```
