# Supplementary material for: Flexible comparative genomics of prokaryotic transcriptional regulatory networks
Source: BMC Genomics. 2020 Dec 16;21(Suppl 5):466. doi: 10.1186/s12864-020-06838-x (PMC7739468; doi:10.1186/s12864-020-06838-x)
Supplement: Supplementary file 3 — Additional file 3. [file 12864_2020_6838_MOESM3_ESM.pdf]

Primer name	Primer sequence (5’- 3’)
plexABvulF	CTTCATGAAATATCTTCTTTTATTGCACATACTTTATACATTAAGCTATTGATTATGGATAATGCGCATCTGACTAGAAAGCAAAAAGAATTCTTCGAGA
plexABvulR	CTCGAAGAATTCTTTTTGCTTTCTAGTCAGATGCGCATTATCCATAATCAATAGCTTAATGTATAAAGTATGTGCAATAAAAGAAGATATTTCATGAAGA
p13210BvulF	ATGACTAAACTTTTTTTGTAAGGAATATATTTTATTTGGGTCTTACATACATTACACATATTTTCTCATCGGAATTACGATGACACGATGTGACACCCCA
p13210BvulR	GGGGTGTCACATCGTGTCATCGTAATTCCGATGAGAAAATATGTGTAATGTATGTAAGACCCAAATAAAATATATTCCTTACAAAAAAAGTTTAGTCATA
p042255BvulF	CTATTTATAGAAGGCTTTTTTATTGGGCTAAATTTTCAATACCTTATATGTATAAAATATGTGTGATGAAAAAAGTTCAGAATCCTAAACGTGGTCGAGA
p042255BvulR	CTCGACCACGTTTAGGATTCTGAACTTTTTTCATCACACATATTTTATACATATAAGGTATTGAAAATTTAGCCCAATAAAAAAGCCTTCTATAAATAGA
p06965BvulF	GCCCATTATTCTCGCTTAACAATGTTTTTGATTTCAGTTATATGTATAATTAATGTATGAAACCAACGACCATATACCGCAAAATAATCCATATTGATAA
p06965BvulR	TATCAATATGGATTATTTTGCGGTATATGGTCGTTGGTTTCATACATTAATTATACATATAACTGAAATCAAAAACATTGTTAAGCGAGAATAATGGGCA
p02350BvulF	TCATGACCTTCTTTATTTGGTGGTTTTAAACTATACACATATTTTGTACATATCAATAGTTGGCTAAATAAATCATGAAAAAGAAGAAGGATTTCGCATA
p02350BvulR	ATGCGAAATCCTTCTTCTTTTTCATGATTTATTTAGCCAACTATTGATATGTACAAAATATGTGTATAGTTTAAAACCACCAAATAAAGAAGGTCATGAA
p01225BvulF	AATTATTGACTTATATAAATATGGGGCGTGTTGTAATTATCATGCACATTTATTTTACATATAGGCGGATATCTATTATCATTCGACATGAAGAAGTTCA
p01225BvulR	GAACTTCTTCATGTCGAATGATAATAGATATCCGCCTATATGTAAAATAAATGTGCATGATAATTACAACACGCCCCATATTTATATAAGTCAATAATTA
p03130RhalF	TTTCGGGTCCATACCACAGTATAAAAAGTTTATTTACATATTTCATACATATATTTTTATGGTTGTTATTCCGCTATCATCAAAGCCAAACGAGGCGCCA
p03130RhalR	GGCGCCTCGTTTGGCTTTGATGATAGCGGAATAACAACCATAAAAATATATGTATGAAATATGTAAATAAACTTTTTATACTGTGGTATGGACCCGAAAA
plexABvulM1F	CTTCATGAAATATCTTCTTTTATTGCAGGTACTTTATACATTAAGCTATTGATTATGGATAATGCGCATCTGACTAGAAAGCAAAAAGAATTCTTCGAGA
plexABvulM1R	CTCGAAGAATTCTTTTTGCTTTCTAGTCAGATGCGCATTATCCATAATCAATAGCTTAATGTATAAAGTACCTGCAATAAAAGAAGATATTTCATGAAGA
plexABvulM2F	CTTCATGAAATATCTTCTTTTATTGCACATACTGGGTTATACATTAAGCTATTGATTATGGATAATGCGCATCTGACTAGAAAGCAAAAAGAATTCTTCA
plexABvulM2R	GAAGAATTCTTTTTGCTTTCTAGTCAGATGCGCATTATCCATAATCAATAGCTTAATGTATAACCCAGTATGTGCAATAAAAGAAGATATTTCATGAAGA

plexABvulM3F	CTTCATGAAATATCTTCTTTTATTGCACATACTTTATAGGTTAAGCTATTGATTATGGATAATGCGCATCTGACTAGAAAGCAAAAAGAATTCTTCGAGA
plexABvulM3R	CTCGAAGAATTCTTTTTGCTTTCTAGTCAGATGCGCATTATCCATAATCAATAGCTTAACCTATAAAGTATGTGCAATAAAAGAAGATATTTCATGAAGA
plexABvulM4F	CTTCATGAAATATCTTCTTTTATTGCACATGGTTTATACATTAAGCTATTGATTATGGATAATGCGCATCTGACTAGAAAGCAAAAAGAATTCTTCGAGA
plexABvulM4R	CTCGAAGAATTCTTTTTGCTTTCTAGTCAGATGCGCATTATCCATAATCAATAGCTTAATGTATAAACCATGTGCAATAAAAGAAGATATTTCATGAAGA
plexABvulM5F	CTTCATGAAATATCTTCTTTTATTGCGCATACTTTATACATTAAGCTATTGATTATGGATAATGCGCATCTGACTAGAAAGCAAAAAGAATTCTTCGAGA
plexABvulM5R	CTCGAAGAATTCTTTTTGCTTTCTAGTCAGATGCGCATTATCCATAATCAATAGCTTAATGTATAAAGTATGCGCAATAAAAGAAGATATTTCATGAAGA
plexABvulM6F	CTTCATGAAATATCTTCTTTTATTGCAGATACTTTATACATTAAGCTATTGATTATGGATAATGCGCATCTGACTAGAAAGCAAAAAGAATTCTTCGAGA
plexABvulM6R	CTCGAAGAATTCTTTTTGCTTTCTAGTCAGATGCGCATTATCCATAATCAATAGCTTAATGTATAAAGTATCTGCAATAAAAGAAGATATTTCATGAAGA
plexABvulM7F	CTTCATGAAATATCTTCTTTTATTGCACGTACTTTATACATTAAGCTATTGATTATGGATAATGCGCATCTGACTAGAAAGCAAAAAGAATTCTTCGAGA
plexABvulM7R	CTCGAAGAATTCTTTTTGCTTTCTAGTCAGATGCGCATTATCCATAATCAATAGCTTAATGTATAAAGTACGTGCAATAAAAGAAGATATTTCATGAAGA
plexABvulM8F	CTTCATGAAATATCTTCTTTTATTGCACAGACTTTATACATTAAGCTATTGATTATGGATAATGCGCATCTGACTAGAAAGCAAAAAGAATTCTTCGAGA
plexABvulM8R	CTCGAAGAATTCTTTTTGCTTTCTAGTCAGATGCGCATTATCCATAATCAATAGCTTAATGTATAAAGTCTGTGCAATAAAAGAAGATATTTCATGAAGA
lexABvulHF	TTCACACAGGAAACAGTACAATGGGCTCTTCCCACCATC
lexABvulHR	TCGACCCGGGGAATTCCGGGTTATGCCACTTTGGCGGTC
M13FpUC	GTTTTCCCAGTCACGAC
M13RpUC	CAGGAAACAGCTATGAC
M13FpUC-DIG	DIG / GTTTTCCCAGTCACGAC
M13RpUC-DIG	DIG / CAGGAAACAGCTATGAC
